# Altered gut microbiota in Rett syndrome

**DOI:** 10.1186/s40168-016-0185-y

**Published:** 2016-07-30

**Authors:** Francesco Strati, Duccio Cavalieri, Davide Albanese, Claudio De Felice, Claudio Donati, Joussef Hayek, Olivier Jousson, Silvia Leoncini, Massimo Pindo, Daniela Renzi, Lisa Rizzetto, Irene Stefanini, Antonio Calabrò, Carlotta De Filippo

**Affiliations:** 1 Department of Computational Biology, Research and Innovation Centre, Fondazione Edmund Mach, Via E. Mach 1, 38010 San Michele all’ Adige, Italy; 2 Centre for Integrative Biology, University of Trento, Via Sommarive 9, 38123 Trento, Italy; 3 Department of Biology, University of Florence, Via Madonna del Piano 6, 50019 Sesto Fiorentino, Florence Italy; 4 Neonatal Intensive Care Unit, University Hospital AOUS, Viale Bracci 16, 53100 Siena, Italy; 5 Child Neuropsychiatry Unit, University Hospital AOUS, Viale Bracci 16, 53100 Siena, Italy; 6 Department of Genomics and Biology of Fruit Crop, Research and Innovation Centre, Fondazione Edmund Mach, Via E. Mach 1, 38010 San Michele all’ Adige, Italy; 7 Department of Experimental and Clinical Biomedical Sciences, Gastroenterology Unit, University of Florence, Viale Morgagni 40, 50139 Florence, Italy; 8 Institute of Biometeorology (IBIMET), National Research Council (CNR), Via Giovanni Caproni 8, I-50145 Florence, Italy

**Keywords:** Gut microbiota, Mycobiota, Rett syndrome, SCFAs, Metataxonomics, Intestinal dysbiosis, Constipation

## Abstract

**Background:**

The human gut microbiota directly affects human health, and its alteration can lead to gastrointestinal abnormalities and inflammation. Rett syndrome (RTT), a progressive neurological disorder mainly caused by mutations in *MeCP2* gene, is commonly associated with gastrointestinal dysfunctions and constipation, suggesting a link between RTT’s gastrointestinal abnormalities and the gut microbiota. The aim of this study was to evaluate the bacterial and fungal gut microbiota in a cohort of RTT subjects integrating clinical, metabolomics and metagenomics data to understand if changes in the gut microbiota of RTT subjects could be associated with gastrointestinal abnormalities and inflammatory status.

**Results:**

Our findings revealed the occurrence of an intestinal sub-inflammatory status in RTT subjects as measured by the elevated values of faecal calprotectin and erythrocyte sedimentation rate. We showed that, overall, RTT subjects harbour bacterial and fungal microbiota altered in terms of relative abundances from those of healthy controls, with a reduced microbial richness and dominated by microbial taxa belonging to *Bifidobacterium*, several Clostridia (among which *Anaerostipes*, *Clostridium XIVa*, *Clostridium XIVb*) as well as *Erysipelotrichaceae*, *Actinomyces*, *Lactobacillus*, *Enterococcus*, *Eggerthella*, *Escherichia/Shigella* and the fungal genus *Candida*.

We further observed that alterations of the gut microbiota do not depend on the constipation status of RTT subjects and that this dysbiotic microbiota produced altered short chain fatty acids profiles.

**Conclusions:**

We demonstrated for the first time that RTT is associated with a dysbiosis of both the bacterial and fungal component of the gut microbiota, suggesting that impairments of MeCP2 functioning favour the establishment of a microbial community adapted to the costive gastrointestinal niche of RTT subjects. The altered production of short chain fatty acids associated with this microbiota might reinforce the constipation status of RTT subjects and contribute to RTT gastrointestinal physiopathology.

**Electronic supplementary material:**

The online version of this article (doi:10.1186/s40168-016-0185-y) contains supplementary material, which is available to authorized users.

## Background

Rett syndrome (RTT; OMIM #312750) is a severe and progressive neurological disorder that almost exclusively affects females with an incidence of ~1:10,000 live births [1]. Loss-of-function mutations of the X-linked methyl-CpG binding protein 2 (*MeCP2*) gene is the major cause (approximately 90 %) of classical cases of RTT while cyclin-dependent kinase-like 5 (*CDKL5*) and forkhead box protein G1 (*FOXG1*) gene mutations represent the remaining 10 % of the cases [2]. MeCP2 is a fundamental mediator of synaptic development and plasticity, and its function is critical in the regulation of synaptic activities during early postnatal development [1]. Different *MeCP2* mutations are also known to correlate with the clinical severity of RTT [3, 4] and the role of MeCP2 in other neurodevelopmental disorders, such as autism, has been demonstrated [5]. RTT subjects develop normally up to 6–18 months of age after which they undergo a period of neurological regression [1]. Microcephaly, dyspraxia, stereotyped hand movements, transient autistic features, respiratory abnormalities, bruxism, seizures and gastrointestinal (GI) dysfunctions are symptoms commonly reported in RTT indicating it as a multisystemic disorder [1]. Among the above-mentioned comorbidities, several epidemiological studies indicated that GI dysfunctions are prevalent through the entire life of RTT subjects [6, 7] with constipation as one of the most frequently reported GI symptoms [6, 7]. Recently, it has been shown that the phenomenon of gut dismotility observed in RTT may arise from impairments in the function of MeCP2 in the enteric nervous system (ENS) [8]. Nevertheless, the direct causes of these GI dysfunctions are still unclear and the role of gut microbiota in host physiology should not be neglected. Indeed, the human gut microbiota plays a crucial role in the function and integrity of the GI tract, maintenance of immune homeostasis and host energy metabolism [9]. Alterations in the composition of commensal bacterial population can lead to chronic inflammation encompassing hyperactivation of T-helper 1 and T-helper 17 immune responses [10], also predisposing individuals to fungal infections [11]. Abnormal immunological response to fungi can in turn contribute to systemic responses including chronic inflammation as observed in inflammatory bowel diseases [12]. Dysbioses of the gut microbiota have been associated with an increasing number of health conditions [13]. A strict relationship between the gut microbiota and the central nervous system (CNS) has been observed, and numerous studies have shown alterations of the gut microbiota in the heterogeneous group of neurological disorders belonging to the autism spectrum disorders (ASDs) [14]. In addition, the gut microbiota may modulate CNS activities through neural, endocrine, metabolic and immune pathways [15] affecting complex physiological and behavioural states of the host [15, 16] so it is possible to hypothesize gut microbiota alterations in RTT as occur in ASDs. Supported by the increasing appreciation of the gut-microbiome-brain axis, we asked whether MeCP2 impairments in RTT might affect also the composition of the gut microbiota resulting in an eventual intestinal dysbiosis in RTT subjects. Indeed, in the case of RTT, it is possible that alterations in the composition of gut microbiota triggered by the neurophysiological changes typical of the disease could contribute to GI abnormalities and be an additional factor relevant to previously observed cytokine dysregulation and systemic inflammation [17, 18]. Here, we characterized for the first time the intestinal microbiota, both bacterial and fungal, in subjects affected by RTT in order to investigate the implication of gut microorganisms and their metabolism on RTT gastrointestinal physiology evaluating also how the constipation status may affect the composition of the gut microbiota in RTT subjects.

## Results

### RTT is associated with mild intestinal inflammation

We analysed the inflammatory status and GI abnormalities in a cohort of 50 RTT subjects by measuring the erythrocyte sedimentation rate (ESR), C-reactive protein (CRP), serum IgA and faecal calprotectin (Additional file 1: Table S1). We found that RTT subjects presented elevated values of ESR (median value 22 mm/h; interquartile range 10–36.5 mm/h; average 26.8 ± 22.4 mm/h) and faecal calprotectin (median value 63.45 μg/g; interquartile range 44–123 μg/g; average 104 ± 97.8 μg/g) compared to a healthy population [19, 20]. Constipation, one of the most common GI symptoms of RTT, was present in 70.8 % of the examined RTT cohort, and it correlated with the titre of serum IgA antibodies (Spearman’s *r* = 0.43, *p* = 0.011) while the levels of serum IgA positively correlated with the ESR values (Spearman’s *r* = 0.462, *p* = 0.011). Constipation, ESR and serum IgA titre correlated with the age of RTT subjects (Spearman’s *r* = 0.35, 0.409 and 0.596, respectively, *p* < 0.05) (Additional file 2: Figure S1, Additional file 3: Table S2). Altogether, these parameters confirmed the presence of a mild GI inflammation in RTT subjects.

### RTT subjects harbour an altered and less diverse gut microbiota

We characterized the bacterial gut microbiota associated with RTT by means of high-throughput sequencing of the V3-V5 region of the 16S rDNA gene. We quantified the bacterial richness within each sample (*alpha*-diversity) of the two groups, RTT subjects and healthy controls (hereinafter termed HC). Three different *alpha*-diversity estimators were used, namely the observed number of OTUs, the Chao1 index and the Shannon entropy index. The bacterial gut microbiota of RTT subjects was significantly less diverse compared to that of HC (*p* < 0.005, Wilcoxon rank-sum test) with all the three estimators used.

**Fig. 1:**
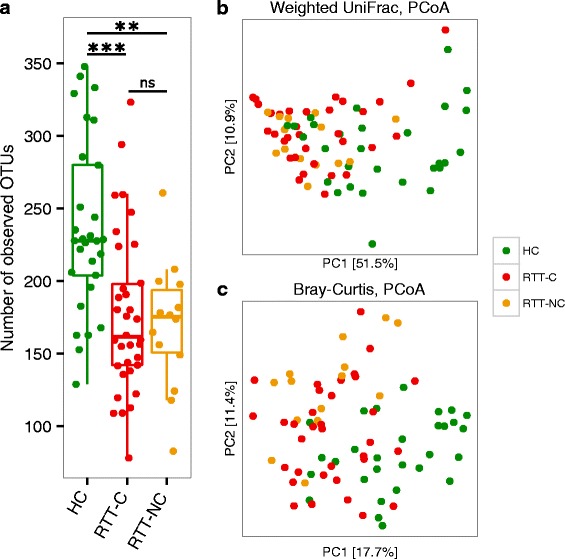
Measures of bacterial diversity. **a** *Alpha*-diversity calculated on the number of observed OTUs; ***p < 0.0001; **p < 0.001; *ns*, not significant; Wilcoxon rank-sum test. **b**, **c** PCoA plots of bacterial *beta*-diversity based on **b** the Weighted UniFrac distance and **c** the Bray-Curtis dissimilarity analysed according to individuals’ health status. Constipated Rett syndrome subjects (RTT-C), non-constipated Rett syndrome subjects (RTT-NC) and healthy controls (HC) are coloured in *red*, *orange* or *green*, respectively

Since constipation affects more than the 70 % of the RTT study cohort, we asked whether the constipation status might be responsible for the differences observed between RTT subjects and HC. The analysis of *alpha*-diversity revealed that, even when analysed separately, both constipated and non-constipated RTT subjects (respectively RTT-C and RTT-NC) harbour a less diverse gut microbiota with respect to HC (*p* < 0.05, Wilcoxon rank-sum test; Fig. 1a and Additional file 3: Figure S2a) while there was no significant difference between RTT-C and RTT-NC (*p* > 0.05, Wilcoxon rank-sum test; Fig. 1a and Additional file 4: Figure S2a). To assess the robustness of these results, we repeated the computation of the *alpha*-diversity using 100 independent rarefactions and for different values of the rarefaction depth. *Alpha* rarefaction curves are reported in the Additional file 5: Figure S3a.

To identify possible differences between the bacterial components of the gut microbiota of RTT subjects and HC, we calculated the *beta*-diversity of the samples using the unweighted and weighted UniFrac distances and the Bray-Curtis dissimilarity. The Principal Coordinates Analysis (PCoA) based on these measures (Fig. 1b and Additional file 4: Figure S2b) revealed that the gut microbiota of RTT subjects was distinct from those of the HC (*p* ≤ 0.003, PERMANOVA) (Table 1). The analysis of *beta*-diversity among HC, RTT-C and RTT-NC revealed significant differences between HC and both RTT-C and RTT-NC (*p* ≤ 0.003, PERMANOVA), but no significant difference was detected when comparing the gut microbiota of RTT-C and RTT-NC (Table 1). Multiple-rarefaction PCoA plots (“jackknifed” PCoA plots, [21]) (Additional file 6: Figure S4a and Additional file 7: Figure S5a) were computed to assess the robustness of bacterial *beta*-diversity analyses, showing that these results hold independently from rarefaction.

**Table 1:** Permutational multivariate analysis of variance (PERMANOVA) tests of the bacterial gut microbiota on the unweighted and weighted UniFrac distances and the Bray-Curtis dissimilarity according to individuals’ health status and constipation

	Metric	*F*	*R* ^2^	*p* value*
HC (*n* = 29) vs RTT (*n* = 50)	Unweighted UniFrac	6.84	0.08	≤0.003
	Weighted UniFrac	15.6	0.16	≤0.003
	Bray-Curtis	6.94	0.08	≤0.003
HC (*n* = 29) vs RTT-C (*n* = 34)	Unweighted UniFrac	5.59	0.08	≤0.003
	Weighted UniFrac	12.1	0.16	≤0.003
	Bray-Curtis	5.39	0.08	≤0.003
HC (*n* = 29) vs RTT-NC (*n* = 14)	Unweighted UniFrac	5.27	0.11	≤0.003
	Weighted UniFrac	8.75	0.17	≤0.003
	Bray-Curtis	5.47	0.11	≤0.003
RTT-C (*n* = 34) vs RTT-NC (*n* = 14)	Unweighted UniFrac	1.31	0.02	0.180
	Weighted UniFrac	1.10	0.02	0.687
	Bray-Curtis	1.76	0.03	0.180

*HC* healthy controls, *RTT-C* constipated Rett syndrome subjects, *RTT-NC* non-constipated Rett syndrome subjects

*Bonferroni corrected *p* values

### *Bifidobacterium* is the hallmark of intestinal dysbiosis in RTT

Since we did not observe population differences in the gut microbiota of RTT subjects in function of the constipation status, only the health condition of the subjects (i.e., healthy or RTT) was considered in the following analyses. To identify the taxa that were differentially represented in HC and RTT subjects, we compared the relative abundances between these two groups at different taxonomic levels.

Phylum level analysis showed that *Actinobacteria* was the most abundant phylum in RTT with a significant increase of its relative abundance in RTT subjects compared to HC (*p* = 0.0017, Wilcoxon rank-sum test; Additional file 8: Table S3 and Additional file 9: Table S4). Furthermore, while *Firmicutes* was the most abundant phylum in HC, we observed a significant decrease of the relative abundance of *Bacteroidetes* in RTT subjects (*p* = 0.002, Wilcoxon rank-sum test; Additional file 8: Table S3 and Additional file 9: Table S4). Indeed, the significant increase of the *Firmicutes*/*Bacteroidetes* ratio, a rough estimator of intestinal dysbiosis, in RTT subjects (median value 3.95) compared to HC (median value 1.64) (*p* = 0.003, Wilcoxon rank-sum test) indicated the presence of an intestinal dysbiosis associated with RTT.

Analysis of the relative abundance of bacterial taxonomic groups at the genus level showed that the ten most abundant genera were *Bifidobacterium* (mean relative abundance, RTT, 36.7 %; HC, 17.2 %), *Bacteroides* (RTT, 12.3 %; HC, 18.3 %), *Faecalibacterium* (RTT, 3.6 %; HC, 9.2 %), *Lachnospiracea incertae sedis* (RTT, 4.6 %; HC, 3.9 %), *Blautia* (RTT, 4.7 %; HC, 3.7 %), *Escherichia/Shigella* (RTT, 5.2 %; HC, 2.4 %), *Alistipes* (RTT, 1.3 %; HC, 4.7 %), *Streptococcus* (RTT, 2.3 %; HC, 2.9 %), *Gemmiger* (RTT, 1.1 %; HC, 3.1 %) and *Ruminococcus* (RTT, 1.5 %; HC, 2.2 %) (Additional file 10: Figure S6 and Additional file 8: Table S3). Comparing the relative abundance of all the genera among the two groups of study, we discovered *Actinomyces*, *Bifidobacterium*, *Clostridium XIVa Eggerthella*, *Enterococcus*, *Erysipelotrichaceae incertae sedis*, *Escherichia/Shigella* and *Megasphaera*, as significantly more abundant in RTT subjects compared to HC while several other bacterial genera usually associated with a healthy human gut were less abundant in RTT subjects compared to HC (*p* < 0.05, Wilcoxon rank-sum test; Additional file 11: Figure S7, Additional file 8: Table S3 and Additional file 9: Table S4).

**Fig. 2:**
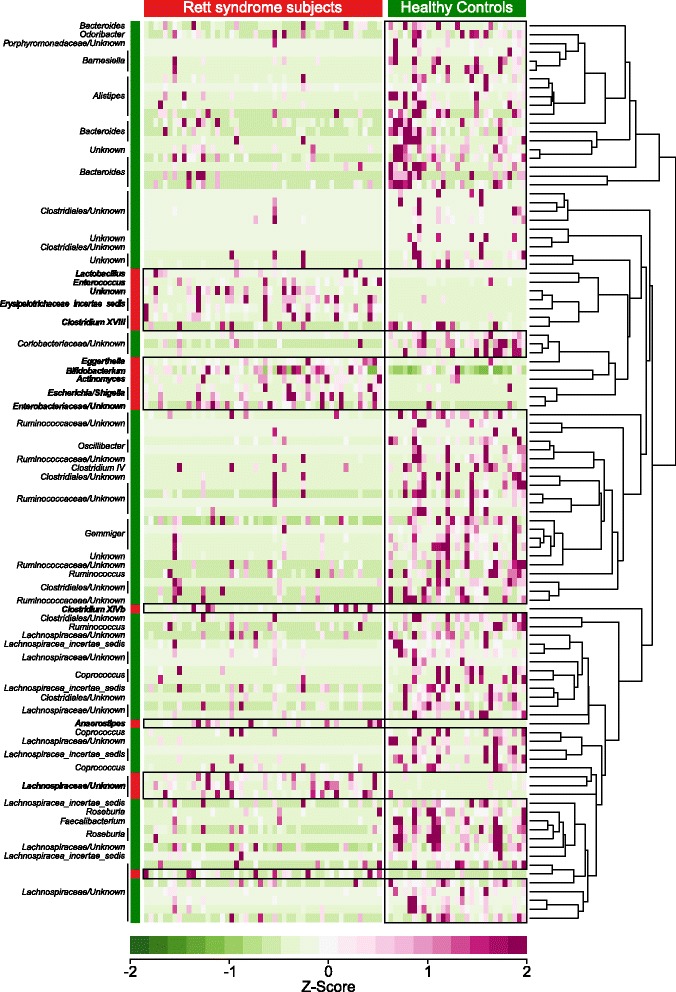
PhyloRelief analysis (RTT vs HC) of bacterial OTUs using the unweighted UniFrac distance. The heat-map shows the relative abundances of the OTUs that are differentially represented in Rett syndrome (RTT) subjects and healthy controls (HC) (PhyloRelief selected clades with FDR-corrected *p* < 0.01, Kruskal-Wallis test). OTUs are classified according to their genus on the left side of the figure. The OTUs more represented in RTT subjects than HC are highlighted in *bold characters*. The ultrametric pruned phylogenetic tree of the OTUs is shown on the right side of the figure. RTT subjects and healthy controls are coloured in *red* and *green*, respectively. Abundances are expressed in terms of their z-score

To define more precisely the taxa that were driving the differentiation of the microbiota of the groups, we performed an analysis based on PhyloRelief [22], a recent phylogenetic-based feature weighting algorithm for metagenomics data. Being independent from a pre-compiled taxonomy, PhyloRelief includes in the analysis unclassified taxa that would be otherwise ignored by other methods. The PhyloRelief analysis confirmed that the *Bifidobacterium* clade was significantly more represented in RTT subjects with respect to HC (Fig. 2). In addition, several OTUs classified as belonging to different members of Clostridia (e.g., *Anaerostipes*, *Clostridium XIVb*, *unknown-Lachnospiraceae*) as well as *Erysipelotrichaceae* (*Clostridium XVIII* and *Erysipelotrichaceae incertae sedis*), *Actinomyces*, *Lactobacillus*, *Eggerthella*, *Enterococcus* and *Enterobacteriaceae* (in particular *Escherichia/Shigella*) were significantly more abundant in the gut microbiota of RTT subjects compared to HC (Fig. 2). Remarkably, the PhyloRelief analysis identified significant differences in the taxa *Anaerostipes, Clostridium XIVb*, *Clostridium XVIII*, *Lactobacillus* and *Clostridium IV* that went undetected by using the Wilcoxon rank-sum test (Additional file 9: Table S4)*.*

**Fig. 3:**
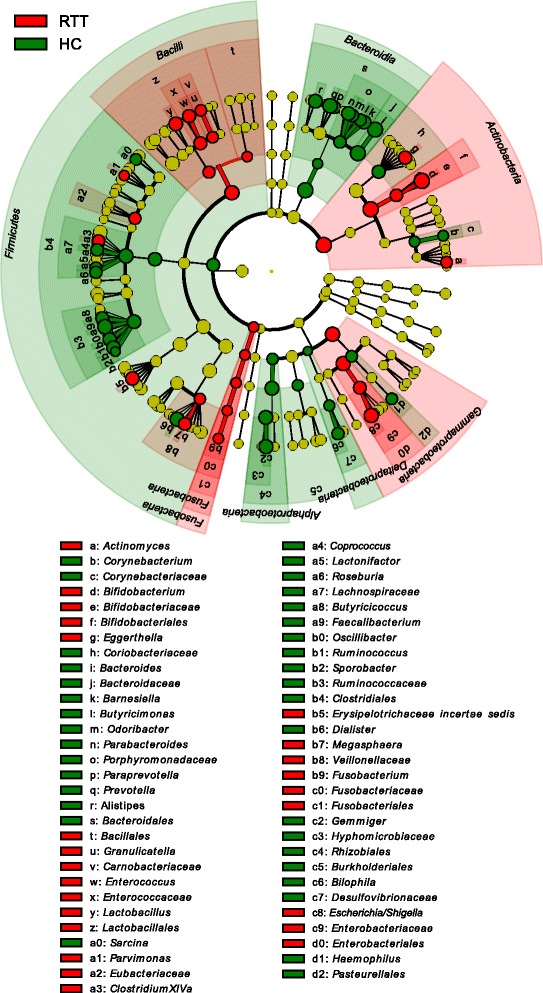
Cladogram showing the most discriminative bacterial clades identified by LEfSe. Coloured regions/branches indicate differences in the bacterial population structure between Rett syndrome (RTT) subjects and healthy controls (HC). Regions in *red* indicate clades that were enriched in RTT subjects compared to those in HC, while regions in *green* indicate clades that were enriched in HC compared to those in RTT subjects

Differentially abundant taxa were further confirmed by LEfSe [23], an algorithm for high-dimensional biomarker discovery which exploits linear discriminant analysis (LDA) to robustly identify features statistically different among classes. Figure 3 shows the most relevant clades identified by LEfSe (logarithmic LDA score > 2.0; see also Additional file 12: Figure S8).

**Fig. 4:**
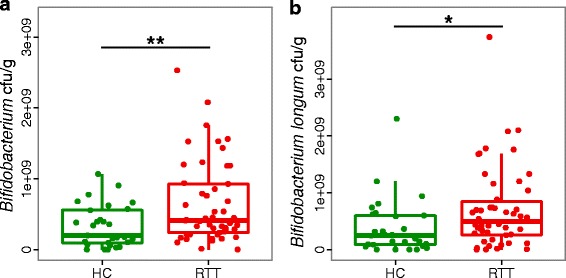
Absolute quantification of *Bifidobacterium*. qPCR analysis of **a** the genus *Bifidobacterium* and **b** the species *Bifidobacterium longum ssp. longum* in Rett syndrome (RTT) subjects versus healthy controls (HC); **p < 0.01, *p < 0.05, Wilcoxon rank-sum test

To evaluate the absolute amount of *Bifidobacterium* and validate their increase in RTT-associated dysbiosis, we performed quantitative PCR analysis (qPCR). We observed that *Bifidobacterium* was twofold more abundant in RTT subjects than in HC (median values 4.08*10^8^ CFU/g and 1.99*10^8^ CFU/g, respectively; *p* = 0.009, Wilcoxon rank-sum test; Fig. 4a). We also noticed that among the *Bifidobacterium* species investigated, *Bifidobacterium longum ssp. longum* was significantly more abundant in RTT subjects compared to HC (median values 4.94*10^8^ CFU/g and 2.44*10^8^ CFU/g respectively; *p* = 0.019, Wilcoxon rank-sum test; Fig. 4b).

### High levels of faecal short chain fatty acids in RTT subjects

**Fig. 5:**
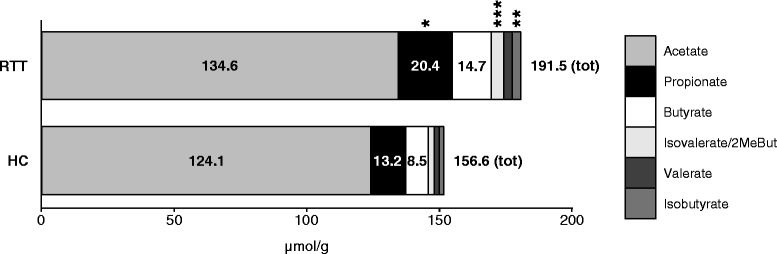
SCFAs faecal content. Bar-plot representation of the median values of faecal SCFAs in Rett syndrome (RTT) subjects and healthy controls (HC); *p < 0.05, **p < 0.005, ***p < 0.0005, Wilcoxon rank-sum test

PICRUSt was used for inference of microbial metabolic pathways [24] in the gut microbiota of RTT subjects and HC (Additional file 13: Table S5). The analysis predicted the enrichment, among others, of carbohydrate and propanoate metabolism in the gut microbiota of RTT subjects, which are metabolic pathways related also to the metabolism of short chain fatty Acids (SCFAs). Since SCFAs are important for colonic health and may act on neuronal physiology [9, 25, 26], we measured the faecal content of SCFAs in our samples by means of GC-MS. We observed that the overall content of SCFAs in RTT subjects’ faeces was higher than in HC (median values RTT, 191.5 μmol/g; HC 156.6 μmol/g). Particularly, we observed that propionate (median values RTT, 20.4 μmol/g; HC 13.2 μmol/g), isovalerate\2-methylbutyrate (median values RTT, 4.7 μmol/g; HC 2.2 μmol/g) and isobutyrate (median values RTT, 3.1 μmol/g; HC 1.6 μmol/g) were significantly more abundant in RTT subjects than HC (*p* < 0.05, Wilcoxon rank-sum test; Fig. 5).

### RTT-associated gut mycobiota shows clear population composition differences compared to HC

The human gut mycobiota has been poorly explored so far, although there is an increasing awareness of its importance in human (patho)physiology [27]. We characterized the gut mycobiota of the study cohort by means of high-throughput sequencing of the ITS1 region of the ribosomal Internal Transcribed Spacer (ITS).

**Fig. 6:**
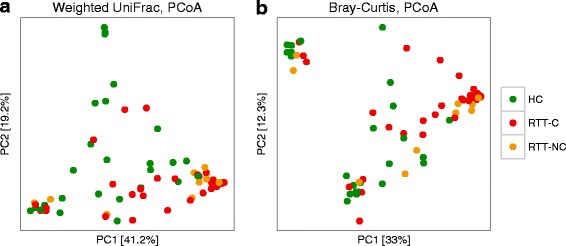
Measures of fungal *beta*-diversity. PCoA plots of fungal *beta*-diversity based on **a** the Weighted UniFrac distance and **b** the Bray-Curtis dissimilarity analysed according to individuals’ health status. Constipated Rett syndrome subjects (RTT-C), non-constipated Rett syndrome subjects (RTT-NC) and healthy controls (HC) are coloured in *red*, *orange* or *green*, respectively

High-quality fungal sequences were detected respectively in 49 out of 50 RTT subjects and 28 out of 29 HC. The analysis of the *alpha*-diversity revealed that the gut mycobiota of RTT subjects, both constipated and non-constipated, was slightly less diverse compared to HC even if no significant differences were observed (*p* > 0.05, Wilcoxon rank-sum test; Additional file 14: Figure S9a). *Alpha* rarefaction curves (using 100 independent rarefactions) are reported in the Additional file 5: Figure S3b. As for the bacterial microbiota, a PERMANOVA analysis on the unweighted, weighted UniFrac distances and Bray-Curtis dissimilarity revealed that the gut mycobiota of RTT subjects was significantly different from that of HC (*p* < 0.05, PERMANOVA; Table 2, Fig. 6 and Additional file 14: Figure S9b), while no significant difference was detected between the gut mycobiota of constipated and non-constipated RTT subjects (Table 2). Multiple-rarefaction PCoA plots (“jackknifed” PCoA plots, [21]) (Additional file 6: Figure S4b and Additional file 7: Figure S5b) computed to assess the robustness of the fungal *beta*-diversity analyses showed that the unweighted UniFrac measure on the gut mycobiota was sensitive to rarefaction (although PERMANOVA *p* values were significant, see Additional file 7: Figure S5b), while the other *beta*-diversity measures support a differentiation between the gut mycobiota of HC and RTT subjects.

**Table 2:** Permutational multivariate analysis of variance (PERMANOVA) tests of the gut mycobiota on the unweighted and weighted UniFrac distances and the Bray-Curtis dissimilarity according to individuals’ health status and constipation

	Metric	*F*	*R* ^2^	*p* value*
HC (*n* = 28) vs RTT (*n* = 49)	Unweighted UniFrac	2.76	0.03	0.006
	Weighted UniFrac	7.45	0.09	≤0.003
	Bray-Curtis	6.84	0.08	0.006
HC (*n* = 28) vs RTT-C (*n* = 33)	Unweighted UniFrac	2.23	0.03	0.018
	Weighted UniFrac	5.60	0.08	0.009
	Bray-Curtis	5.82	0.08	≤0.003
HC (*n* = 28) vs RTT-NC (*n* = 14)	Unweighted UniFrac	2.00	0.04	0.036
	Weighted UniFrac	5.95	0.12	≤0.003
	Bray-Curtis	4.59	0.10	0.006
RTT-C (*n* = 33) vs RTT-NC (*n* = 14)	Unweighted UniFrac	1.01	0.02	1.000
	Weighted UniFrac	1.27	0.02	0.837
	Bray-Curtis	1.00	0.02	1.000

HC healthy controls, *RTT-C* constipated Rett syndrome subjects, *RTT-NC* non-constipated Rett syndrome subjects

*Bonferroni corrected *p* values

### The genus *Candida* predominates in the altered gut mycobiota of RTT subjects

Metataxonomics analysis of the gut mycobiota led to the identification of 77 fungal taxa unambiguously classified to the genus level and 19 taxa only partially classified. The ten most abundant annotated fungal genera were *Candida* (RTT 61.3 %; HC 25.5 %), *Penicillium* (RTT 13.5 %; HC 19.4 %), *Aspergillus* (RTT 7.3 %; HC 6.5 %), *Malassezia* (RTT 3.5 %; HC 4.5 %), *Debaryomyces* (RTT 1.7 %; HC 5.5 %), *Mucor* (RTT 1.1 %; HC 4.6 %), *Eremothecium* (RTT 0.07 %; HC 3.7 %), *Pichia* (RTT 0.1 %; HC 3.5 %), *Cyberlindnera* (RTT 0.5 %; HC 1.7 %), and *Trichosporon* (RTT 1.3 %; HC 0.007 %) (Additional file 15: Figure S10). The relative abundance of the genus *Candida* was significantly higher in RTT subjects than HC (*p* = 0.002, Wilcoxon rank-sum test; Additional file 16: Figure S11). Remarkably, we detected sequences belonging to the single-cell protozoa *Blastocystis* in different healthy controls (in 24.1 % of the inspected healthy individuals) while this genus was present only in one RTT subject. *Blastocystis* is an important eukaryote of the GI tract of healthy individuals [28] being less common in subjects affected by irritable bowel syndrome and inflammatory bowel diseases [29]. The reported high relative abundance of the genus *Candida* in the gut mycobiota of RTT subjects was further confirmed by LEfSe analysis (Additional file 17: Figure S12).

## Discussion

Our study identified a clear dysbiosis of the fungal and bacterial gut microbiota in individuals affected by RTT, a neurological disorder also associated with gastrointestinal symptoms and systemic inflammation. The elevated values of calprotectin and ESR measured in RTT subjects correlating also with the titre of serum IgA antibodies indicated the occurrence of an intestinal sub-inflammatory status, in line with previous indication of a pro-inflammatory status in *MeCP2*-related RTT [18]. A state of intestinal inflammation is also related to loss of intestinal barrier function and the subsequent translocation of pathobionts that may induce systemic responses [10]. RTT subjects displayed on average a lower bacterial gut microbiota richness and diversity compared to HC. We observed a significant increase in the *Firmicutes*/*Bacteroidetes* ratio in RTT subjects due to a reduction of the relative abundance of *Bacteroidetes* in these subjects. An increased *Firmicutes*/*Bacteroidetes* ratio has recently been reported also in children affected by autism [30], and treatment with *Bacteroides fragilis* has been shown to restore autism-related behavioural and GI abnormalities in a mouse model of neurodevelopmental disorders [31].

An in-depth analysis of bacterial taxa revealed that the relative abundances of *Bifidobacterium* and several Clostridia, i.e. *Anaerostipes*, *Clostridium XIVa*, and *Clostridium XIVb*, as well as *Erysipelotrichaceae* (*Clostridium XVIII* and *Erysipelotrichaceae incertae sedis)*, *Actinomyces*, *Eggerthella*, *Enterococcus*, *Escherichia/Shigella* and *Lactobacillus*, were significantly higher in RTT subjects than in HC. Bifidobacteria are well recognized as health-promoting bacteria [32], with potential probiotic properties [33], and have been rarely associated with pathological states [34, 35]. Measuring the absolute abundances of the most common intestinal *Bifidobacterium* species by qPCR, we found that *Bifidobacterium longum ssp. longum* was the most abundant in RTT subjects, with absolute abundance twofold higher than in HC. The reported high abundance of *Bifidobacterium* in RTT subjects could indicate *Bifidobacterium* adaptation to the GI niche associated with RTT.

In line with our results, various studies on the gut microbiota of ASDs subjects reported also the increased incidence of Clostridia [36, 37], one of the most abundant Gram-positive bacteria known to reside in the human gut. Moreover, *Erysipelotrichaceae*, *Lactobacillus* and *Escherichia/Shigella* resulted to be enriched in ASDs [38, 39] in concomitance with a reduction of *Prevotella* [39], consistently with our observations. *Prevotella* is an important member of the human gut microbiota involved in the maintenance of the microbial community structure [40] while *Escherichia/Shigella* genera may exert pro-inflammatory activities and are abundant in subjects with active states of intestinal inflammation [41, 42]. However, given the lack of a consensus [14] and the methodological differences, it is difficult to draw general conclusions by directly comparing the results of the different studies on ASDs’ gut microbiota.

It is well known that perturbations in the composition of commensal bacteria can predispose individuals to fungal infections [43]. We observed a dysbiotic gut mycobiota associated with RTT characterized by an altered community structure dominated by the genus *Candida. Candida* is one of the most common fungal commensals of the GI tract [28] but bacterial dysbiosis can shift *Candida* commensalism to pathogenesis, leading to extended infections and candidiasis [44]. It was also observed that the proportion of opportunistic pathogenic fungi, including *Candida*, increases in a mouse model of intestinal inflammation [12].

The herein described intestinal dysbiosis could be associated with changes in gut metabolite profiles consequently affecting the RTT gastrointestinal physiology. *Bifidobacterium* as well as *Anaerostipes*, *Clostridium XIVa*, *Clostridium XIVb* and *Clostridium XVIII* are known producer of SCFAs as fermentation end-products of carbohydrates and proteins [45]. Also, the lactic-acid bacteria *Lactobacillus* and *Enterococcus* can sustain the production of SCFAs through cross-feeding mechanisms that involves lactate-utilizing gut bacteria [46]. We observed that the faecal content of SCFAs in RTT subjects was significantly enriched in propionate, isobutyrate and isovalerate\2-methylbutyrate. Non-physiological high levels of SCFAs in the gut could contribute to GI symptoms, including the constipation status (which affect more of the 70 % of this RTT study cohort), through the alteration of goblet cell mucin discharge [47] and the inhibition of smooth muscle contraction in the colon [48] mediated by the release of the peptide YY from enteroendocrine cells [49]. Also, prolonged exposure to protein-derived SCFAs (among which isobutyrate, 2-methylbutyrate and isovalerate) and other protein fermentation products, such as ammonia, phenolic compounds or *p*-cresol, may affect the metabolism and the physiology of colonocytes [50]. Remarkably, enteric SCFAs, principally propionate and butyrate, can modulate gene expression, brain function and behaviour, affecting neurotransmitter systems, neuronal cell adhesion, inflammation, oxidative stress, lipid metabolism and mitochondrial function in rat and in vitro cell models of ASDs [51, 52].

Taken together, these observations suggest that functional impairments of MeCP2 favour the establishment of both constipation and adaptation of an intestinal dysbiotic microbial community that may reinforce the constipation status through non-physiological levels of SCFAs. Nevertheless, we are not able to infer the consequentiality of the two phenomenons, i.e. constipation or dysbiosis. The SCFAs produced by the gut microbiota are thus potentially implicated in the chronic constipation often associated with RTT, yet their role in the pathophysiology of RTT remains elusive. Indeed, the high faecal content of SCFAs in RTT subjects could be also related to a reduced intestinal absorption of these and other metabolites in the gut [53], or to increased liberation of SCFAs due to fibre retention in a costive gut. The establishment of intestinal dysbiosis, both at bacterial and fungal level, may reinforce, rather than determine, constipation, which is one of the most common GI problems in RTT. It is worth noting that the constipation status correlates with age suggesting that constipation and intestinal dysbiosis could be temporally connected with the progression of the disease. Furthermore, the reduction of the mucin layer due to the inhibition of goblet cells induced by the high levels of SCFAs might trigger an immunological response that might boost ERS values, serum IgA and the levels of calprotectin simplifying the putative translocation through the intestinal barrier of pathobionts equipped with proper virulence factors, such as *Candida* and *Escherichia/Shigella*, and overall contributing to systemic inflammation and cytokine dysregulation.

## Conclusions

Here, we demonstrated for the first time that RTT is characterized by a dysbiotic bacterial and fungal microbiota showing an overall reduction of the microbial richness and diversity as well as an altered composition of the microbial community structure in RTT subjects. In particular, the increase in the relative abundance of *Bifidobacterium*, Clostridia and *Candida* drives the dysbiotic state associated with RTT. We hypothesize that impairments of MeCP2 functioning promote the establishment of a dysbiotic intestinal microbial community that, in turn, could affect RTT gastrointestinal physiopathology through altered SCFAs production, reinforcing the constipation status itself and favouring inflammation and cytokine dysregulation. Due to the importance that our findings might have in the design of potential therapeutic interventions aimed at gastrointestinal relief in RTT, we are planning to further investigate the gut microbiota dynamics during the progression of the disease in a *MeCP2*-null mouse model applying specific probiotics and prebiotics treatments.

## Methods

### Study participants and sample handling and collection

We recruited 50 female subjects with clinical diagnosis of RTT (average age 12 ± 7.3), genotyped for *MeCP2* and *CDKL5* gene mutations (Additional file 1: Table S1) and 29 age-matched healthy subjects as controls (average age 17 ± 9.6) (Additional file 18: Table S6). RTT subjects with clinically evident inflammatory conditions (i.e. upper respiratory tract infection, pneumonia, urinary infection, stomatitis and periodontal inflammation), either acute or chronic, were excluded. A “compressed” clinical severity score (CSS) was attributed to RTT subjects following thirteen criteria: regression, microcephaly, somatic growth, independent sitting, ambulation, hand use, scoliosis, language, non-verbal communication, respiratory dysfunction, autonomic symptoms, stereotypies and seizures [4]. ESR, CRP and serum IgA levels were assessed as markers of inflammation or GI abnormalities. Gastrointestinal symptoms (i.e. constipation) and intestinal inflammation (i.e. faecal calprotectin levels) [54] were also assessed (Additional file 1: Table S1). The diagnosis of constipation was defined according to Rome III criteria [55]. Stool samples from enrolled subjects were collected, aliquoted as it is and stored at −80 °C until analysis. All subjects of this study were under a Mediterranean-based diet and no antibiotics, probiotics or prebiotics have been taken in the 3 months prior the sample collection. The study was approved by the institutional review board of the Siena University Hospital (AOUS, Siena, Italy), and all enrolled subjects or tutors gave written informed consent in accordance with the sampling protocol approved by the local Ethical Committee (No. 2012-005021-76).

### Faecal calprotectin assay

Calprotectin determination was performed by using a polyclonal antibody in an enzyme-linked immunosorbent assay (Calprest, Eurospital, Trieste, Italy).

Briefly, frozen stool samples were thawed at room temperature; 100 mg of faeces (wet weight) were weighed and placed in a disposable screw cap-tube containing the extraction buffer (weight/volume 1:50). Samples were then mixed vigorously for 30 s, homogenized for 25 min on a shaker, and centrifuged for 20 min at 10,000*g* at room temperature; 0.5 ml of clear extract supernatant was transferred to new tubes and stored at –80 °C. Finally, samples were diluted 1:50, and absorbance was measured at 405 nm. According to the manufacturer’s instructions, normal values were considered <50 μg/g of calprotectin per faecal sample.

### DNA extraction, PCR amplification of the V3-V5 region of bacterial 16S rDNA and of the ITS1 region of fungal rDNA

Total DNA extraction from faecal samples (250 mg, wet weight) was performed using the FastDNA™ SPIN Kit for Feces (MP Biomedicals, Santa Ana, CA, USA) following manufacturer’s instructions. DNA integrity and quality were checked on 1 % agarose gel TAE 1X and quantified with a NanoDrop® spectrophotometer. For each DNA sample, 16S rRNA gene was amplified using fusion primer set specific for V3-V5 hypervariable regions (357F: 5′-TCCTACGGGAGGCAGCAG-3′ and 937R: 5′-TGTGCGGGCCCCCGTCAATT-3′) containing adaptors, key sequence and barcode (Multiple IDentifier) sequences as described by the 454 Sequencing System Guidelines for Amplicon Experimental Design (Roche, Basel, Switzerland).

PCR reactions were performed using the FastStart High Fidelity PCR system (Roche, Basel, Switzerland) according to the following protocol: 5 min at 95 °C, 25 cycles of 30 s at 95 °C, 30 s at 58 °C and 1 min at 72 °C, followed by a final extension of 8 min at 72 °C. The PCR reaction mix contained 1X FastStart High Fidelity PCR buffer 1.8 mM MgCl_2_, 200 μM of dNTPs, 0.4 μM of each primer (Eurofins, PRIMM, Milano, Italy), 2.5 U of FastStart High Fidelity Polymerase Blend and 10 ng of gDNA as template. For ITS1 amplicon sequencing, fusion primer sets were designed as described above coupled with forward primer 18SF (5′-GTAAAAGTCGTAACAAGGTTTC-3′) and reverse primer 5.8S1R (5′-GTTCAAAGAYTCGATGATTCAC-3′) [56] specific for fungal ITS1 rDNA region. The PCR reaction mix contained 1X FastStart High Fidelity PCR buffer, 2 mM MgCl_2_, 200 μM of dNTPs, 0.4 μM of each primer (PRIMM, Milano, Italy), 2.5 U of FastStart High Fidelity Polymerase Blend and 100 ng of gDNA as template. Thermal cycling conditions used were 5 min at 95 °C, 35 cycles of 45 s at 95 °C, 45 s at 56 °C and 1.30 min at 72 °C followed by a final extension of 10 min at 72 °C. All PCR experiments were carried out in triplicates using a Veriti® Thermal Cycler (Applied Biosystems, Foster City, CA, USA).

### Library construction and pyrosequencing

The PCR products obtained were analysed by gel electrophoresis and cleaned using the AMPure XP beads kit (Beckman Coulter, Brea, CA, USA) following the manufacturer’s instructions, quantified via quantitative PCR using the Library quantification kit—Roche 454 titanium (KAPA Biosystems, Boston, MA) and pooled in equimolar way in a final amplicon library. The 454 pyrosequencing was carried out on the GS FLX+ system using the XL+ chemistry following the manufacturer’s recommendations (Roche, Basel, Switzerland).

### qPCR analysis

Amplifications of *Bifidobacterium sp.* 16S rDNA gene were performed in triplicate for each sample using the KAPA SYBR® FAST qPCR Kit Optimized for LightCycler® 480 (Kapa Biosystems, Inc., Wilmington, MA, USA) and the LightCycler® 480 II instrument (Roche, Basel, Switzerland) with primers and protocols described previously [57, 58]. The PCR reaction mix contained 1X KAPA SYBR FAST qPCR Master Mix, 0.2 μM of each *Bifidobacterium*-specific primer and 10 ng of gDNA as template. For quantification of each *Bifidobacterium* species, we constructed a seven-point standard curve consisting in tenfold serial dilutions of gDNA extracted from a pure culture at known concentration. For quantification of the genus *Bifidobacterium*, we used the strain *Bifidobacterium animalis ssp. lactis* BB12. The following *Bifidobacterium* type strains were used for the species-specific quantification: *B. angulatum* ATCC27535, *B. adolescentis* ATCC15703, *B. animalis ssp. lactis* ATCC15705, *B. bifidum* DSM20456, *B. breve* ATCC15700, *B. dentium* ATCC27534, *B. longum ssp. infantis* ATCC15697, *B. longum ssp. longum* ATCC15707 and *B. pseudocatenulatum* ATCC27917. Amplification specificity of target gene was checked by melting curve analysis. Efficiency and reliability of PCR amplifications were calculated.

### SCFAs analysis

Frozen faecal samples (∼150 mg, wet weight) were diluted 1:10 in sterile PBS 1 M (pH 7.2) and centrifuged at 13.000*g* for 5 min. Supernatants were then filtered using a 0.2-μm polycarbonate syringe filter and acidified by the addition of one volume of HCl 6 M to three volumes of sample. After 10-min incubation at room temperature, samples were centrifuged at 13.000*g* for 5 min. One volume of 10 mM 2-ethylbutyric acid was added to four volumes of sample as internal standard. Calibration was done using standard solutions of acetate, propionate, butyrate, isobutyrate, 2-methyl-butyrate (2-MeBut), valerate and isovalerate in acidified water (pH 2). Standard solutions containing 50, 20, 10, 5, 1 and 0.5 mM of each external standard were used.

Analysis was performed using a TRACE™ Ultra Gas Chromatograph (Thermo Scientific, Waltham, MA, USA) coupled to a TSQ Quantum GC mass spectrometric detector (Thermo Scientific, Waltham, MA, USA). SCFAs were separated using a Restek Stabilwax-DA (30 m × 0.25 mm; 0.25-μm film thickness) (Restek corp., Bellafonte, PA, USA). The injected sample volume was 1 μl in split mode with a ratio of 10:1. The initial oven temperature was at 90 °C and maintained for 0.5 min and then increased 20 °C/min to 240 °C. The carrier gas helium was delivered at a flow rate of 1 ml min^−1^. The temperatures of the inlet, transfer line and electron impact (EI) ion source were set at 280, 250 and 250 °C, respectively. The electron energy was 70 eV, and the mass spectral data was collected in a full scan mode (m/z 30–200).

### Data analysis

Pyrosequencing resulted in a total of 2,227,864 reads for 16S rDNA with a mean of 27,987 ± 5782 sequences per sample and 1,678,227 reads for ITS1 region with a mean of 21,118 ± 13,270 sequences per sample. Raw 454 files were demultiplexed using the Roche’s sff file software and submitted to the European Nucleotide Archive with accession number PRJEB12607. Sample accession IDs and metadata are available in Additional file 18: Table S6. Reads were pre-processed using the MICCA pipeline (http://www.micca.org) [59]. Forward and reverse primer trimming and quality filtering were performed using micca-preproc. De novo sequence clustering, chimera filtering and taxonomy assignment were performed by micca-otu-denovo: operational taxonomic units (OTUs) were assigned by clustering the sequences with a threshold of 97 % pairwise identity, and their representative sequences were classified using the RDP classifier version 2.7 on 16S rDNA data and using the RDP classifier version 2.8 [60] against the UNITE fungal ITS database [61] on ITS1 data. Template-guided multiple sequence alignment (MSA) was performed using PyNAST [62] (v. 0.1) against the multiple alignment of the Greengenes [63] database (release 13_05) filtered at 97 % similarity for bacterial sequences and through de novo MSA using T-Coffee [64] for fungal sequences. Fungal taxonomy assignments were also manually curated using BLASTn against the GenBank’s database for accuracy. High-quality fungal sequences have been also manually filtered out for sequences belonging to *Agaricomycetes* (unlikely to be residents of the human gut due to their ecology) [65]. Samples with less than 1000 reads have been excluded from the analysis. The phylogenetic trees were inferred using micca-phylogeny [66]. Sampling heterogeneity was reduced rarefying samples at 90 % of the less abundant sample (16S data) and at the depth of the less abundant sample (ITS1 data). *Alpha*- (within-sample richness) and *beta*-diversity (between-sample dissimilarity) estimates were computed using the phyloseq R package [67]. Multiple-rarefaction PCoA plots (“jackknifed” PCoA plots, [21]) were computed to assess the robustness of the *beta*-diversity analyses. Permutational MANOVA (PERMANOVA) was performed on the UniFrac distances and Bray-Curtis dissimilarity using the adonis() function of the *vegan* R package with 999 permutations, and *p* values were corrected using the Bonferroni correction [68]. The non-parametric Wilcoxon rank-sum test was used for the comparison of relative abundances of microbial taxa between groups, and the resulting *p* values were corrected for multiple testing controlling the false discovery rate [69] at all taxonomic levels taken into account. Further identification of taxa differentially distributed in case/control groups was obtained by PhyloRelief, a phylogenetic-based feature weighting algorithm for metagenomics data. This method unambiguously groups taxa into clades without relying on a precompiled taxonomy and accomplishes a ranking of the clades according to their contribution to the sample differentiation [22]. Linear discriminant effect size analysis (LEfSe) [23] with default parameters was performed to find taxonomic clades differentially represented between RTT subjects and HC. LEfSe combines Kruskal-Wallis test and Wilcoxon rank-sum tests with linear discriminant analysis (LDA). LEfSe ranks features by effect size, putting at the top features that explain most of the biological difference. In order to investigate the microbial metabolic potential of the gut microbiota in healthy controls and RTT subjects, we applied PICRUSt (Phylogenetic Investigation of Communities by Reconstruction of Unobserved States) [24], a computational approach used to predict the functional composition of a metagenome using marker gene data and a database of reference genomes. PICRUSt uses an extended ancestral-state reconstruction algorithm to predict which gene families are present and then combines gene families to estimate the composite metagenome starting from the taxonomic composition estimated from 16S rDNA data. Starting from a table of OTUs with associated Greengenes identifiers, we obtained the final output from metagenome prediction as an annotated table of predicted gene family counts for each sample, where the encoded function of each gene family be orthologous groups or other identifiers such as KEGG orthologues. Spearman’s correlation tests for each correlation were computed using the *psych* R package [70]. All statistical analyses were performed using R [71].

## Abbreviations

ASDs, autism spectrum disorders; CDKL5, cyclin-dependent kinase-like 5; CNS, central nervous system; CRP, C-reactive protein; CSS, clinical severity score; ENS, enteric nervous system; ESR, erythrocyte sedimentation rate; FDR, false discovery rate; GC-MS, gas chromatography–mass spectrometry; GI, gastrointestinal; HC, healthy controls; ITS, internal transcribed spacer; LDA, linear discriminant analysis; LEfSe, linear discriminant effect size analysis; MeCP2, methyl-CpG binding protein 2; OTU, operational taxonomic unit; PCoA, principal coordinates analysis; PERMANOVA, permutational multivariate analysis of variance; PICRUSt, phylogenetic investigation of communities by reconstruction of unobserved states; qPCR, quantitative PCR; RTT, Rett syndrome; RTT-C, constipated Rett syndrome subjects, RTT-NC, non-constipated Rett syndrome subjects; SCFAs, short chain fatty acids.

## Additional files

Additional file 1: Table S1. Characteristics of study participants affected by Rett syndrome. (DOCX 24 kb) Additional file 2: Figure S1. Correlation plots of clinical data from RTT subjects. Significant positive correlations were observed among age, IgA and ESR. (PDF 37 kb) Additional file 3: Table S2. Spearman’s correlation analysis among RTT clinical data. (DOCX 14 kb) Additional file 4: Figure S2. Measures of bacterial diversity. a) *Alpha*-diversity estimated on the Chao1 estimator and the Shannon entropy; ***, *p* < 0.001; **, *p* < 0.01; *, *p* < 0.05; Wilcoxon rank-sum test. b) PCoA plot based on the unweighted UniFrac distance among samples analysed according to individuals’ health status. Constipated Rett syndrome subjects (RTT-C), non-constipated Rett syndrome subjects (RTT-NC) and healthy controls (HC) are coloured in red, orange or green, respectively. (PDF 58 kb) Additional file 5: Figure S3.
*Alpha*-diversity rarefaction curves. The plot shows the *alpha*-diversity for HC, RTT-C and RTT-NC averaged over 100 independent rarefactions as a function of the rarefaction depth for a) the bacterial gut microbiota and b) the fungal gut microbiota. The points in the curves are the averages, while the whiskers represent the standard deviations. (PDF 47 kb) Additional file 6: Figure S4. Multiple-rarefaction PCoA plots. Each PCoA replicate was optimally superimposed by Procrustes analysis on the master PCoA scatter plot (used in the main text). Points represent the average location of 100 rarefaction replicates. Ellipses show the 95 % confidence region assuming a multivariate normal distribution. a) PCoA plots of bacterial *beta*-diversity based on the unweighted and weighted UniFrac distances and the Bray-Curtis dissimilarity analysed according to individuals’ health status; b) PCoA plots of fungal *beta*-diversity based on the unweighted and weighted UniFrac distances and the Bray-Curtis dissimilarity analysed according to individuals’ health status. Constipated Rett syndrome subjects (RTT-C), non-constipated Rett syndrome subjects (RTT-NC) and healthy controls (HC) are coloured in red, orange or green, respectively. (PDF 185 kb) Additional file 7: Figure S5. PERMANOVA *p* values distribution. For each rarefaction replicate (n = 100), a PERMANOVA test was conducted to assess the robustness of the results over the rarefaction. a) PERMANOVA *p* values distributions of bacterial *beta*-diversity based on the unweighted and weighted UniFrac distances and the Bray-Curtis dissimilarity analysed according to individuals’ health status; b) PERMANOVA *p* values distributions of fungal *beta*-diversity based on the unweighted and weighted UniFrac distances and the Bray-Curtis dissimilarity analysed according to individuals’ health status. (PDF 27 kb) Additional file 8: Table S3. Mean relative abundance (%) ± standard deviation (SD) of bacterial OTUs at the phylum and genus levels in Rett syndrome (RTT) subjects and healthy controls (HC). (DOCX 32 kb) Additional file 9: Table S4. Wilcoxon rank-sum test comparison of bacterial relative abundances at phylum and genus levels. (DOCX 22 kb) Additional file 10: Figure S6. Genus level relative abundances of the bacterial gut microbiota of healthy controls (HC) and Rett syndrome (RTT) subjects. (PDF 5470 kb) Additional file 11: Figure S7. Bacterial taxa which relative abundances were significantly different (*p* < 0.05; Wilcoxon rank-sum test) between healthy controls (HC) and Rett syndrome (RTT) subjects. (PDF 319 kb) Additional file 12: Figure S8. LDA scores of the most discriminant bacterial taxa identified by LEfSe. Positive and negative LDA scores indicate the taxa enriched in healthy controls (HC) and Rett syndrome (RTT) subjects, respectively. (PDF 62 kb) Additional file 13: Table S5. Statistics of the significantly different metabolic pathways (KEGG categories) inferred with PICRUSt in the gut microbiota of healthy controls (HC) and Rett syndrome (RTT) subjects (Welch’s *t* test, *p* < 0.05 FDR-corrected) from 16S rDNA data. (DOCX 26 kb) Additional file 14: Figure S9. Measures of fungal diversity. a) Three estimators of *alpha*-diversity have been calculated: the number of observed OTUs, the Chao1 estimator and the Shannon entropy; b) PCoA plot based on the unweighted UniFrac distance among samples analysed according to individuals’ health status. Constipated Rett syndrome subjects (RTT-C), non-constipated Rett syndrome subjects (RTT-NC) and healthy controls (HC) are coloured in red, orange or green, respectively. (PDF 63 kb) Additional file 15: Figure S10. Genus level relative abundances of the fungal gut microbiota of healthy controls (HC) and Rett syndrome (RTT) subjects. (PDF 2981 kb) Additional file 16: Figure S11.
*Candida* relative abundance in the gut microbiota of healthy controls (HC) and Rett syndrome (RTT) subjects (*p* < 0.05; Wilcoxon rank-sum test). (PDF 20 kb) Additional file 17: Figure S12. a) LDA scores of the most discriminant fungal taxa identified by LEfSe. Positive and negative LDA scores indicate the taxa enriched in healthy controls (HC) and Rett syndrome (RTT) subjects, respectively. b) Cladogram showing the most discriminative fungal clades identified by LEfSe. Coloured regions/branches indicate differences in the fungal population structure between Rett syndrome (RTT) subjects and healthy controls (HC). Regions in red indicate clades that were enriched in RTT subjects compared to those in HC, while regions in green indicate clades that were enriched in HC compared to those in RTT subjects. (PDF 116 kb) Additional file 18: Table S6. Correspondences among deposited metagenomics data and samples, unrarefied OTU tables and taxonomic classifications of the 16S and ITS1 datasets. (XLSX 449 kb) Additional file 19. MICCA pipelines used for the analysis of the 16S and ITS1 datasets. (PDF 7 kb)
