# Supplementary material for: Altered gut microbiota in Rett syndrome
Source: Microbiome. 2016 Jul 30;4:41. doi: 10.1186/s40168-016-0185-y (PMC4967335; doi:10.1186/s40168-016-0185-y)
Supplement: Additional file 1: Table S1. — Characteristics of study participants affected by Rett syndrome. (DOCX 24 kb) [file 40168_2016_185_MOESM1_ESM.docx]

**Supplementary Table 1:** Characteristics of study participants affected by Rett syndrome.

| Sample ID | Age | MECp2 Nt mutation* | MECp2 aa mutation* | type of MECp2 mutation* | Domain change location* | Clinic phenotype^#^ | CSS | Constipation | ESR (mm/h) | IgA (mg/ml) | CRP (mg/100ml) | Calprotectin (μg/g) |
| --- | --- | --- | --- | --- | --- | --- | --- | --- | --- | --- | --- | --- |
| RTT4 | 25 | c.747_751dup5 | p.P251fs | Frameshift in/del | TRD | intermediate | 7 | yes | 55 | 128 | <0.35 | 116.9 |
| RTT9 | 3 | deletion exon 3. 4a. 4b | deletion exon 3. 4a. 4b | Large deletion | Large deletion | intermediate | 6 | no | NA | 52 | <0.35 | 57.3 |
| RTT10 | 21 | c.1163_1197del35 | p.P388fs | Frameshift in/del | C-term | intermediate | 7 | yes | 74 | 372 | 0.69 | 171.4 |
| RTT34 | 9 | c.502C>T | p.R168X | Nonsense | Inter-domain region | intermediate | 8 | yes | 49 | 171 | 0.92 | 110.1 |
| RTT35 | 7 | c.1072_1186del113 | Large deletion | Large deletion | C-term | intermediate | 7 | no | 13 | 9.9 | 0.85 | 64.3 |
| RTT38 | 7 | deletion exon 4 | deletion exon 4 | Large deletion | Large deletion | mild | 4 | yes | 21 | 122 | <0.35 | 21.9 |
| RTT39 | 17 | Large deletion | Large deletion | Large deletion | Large deletion | intermediate | 8 | yes | 9 | 253 | <0.35 | 296.1 |
| RTT43 | 13 | c.473C>T | p.T158M | Missense | MBD | severe | 10 | no | 39 | 215 | 2.39 | 50.5 |
| RTT51 | 7 | c.397C>T | p.R133C | Missense | MBD | intermediate | 7 | no | 12 | 171 | <0.35 | 72.1 |
| RTT53 | 15 | del.MECp2 | del.MECp2 | Large deletion | Large deletion | severe | 9 | yes | 40 | 381 | <0.35 | 28.8 |
| RTT72 | 31 | c.880C>T | p.R294X | Nonsense | TRD | severe | 9 | yes | 4 | 22 | <0.35 | 379.8 |
| RTT80 | 4 | c.473C>T | p.T158M | Missense | MBD | NA | NA | no | 5 | 54 | <0.35 | 44.9 |
| RTT91 | 24 | c.916C>T | p.R306C | Missense | TRD | severe | 11 | yes | NA | 317 | 0.55 | 41.1 |
| RTT97 | 19 | c.547G>C | p.G183R | Missense | Inter-domain region | intermediate | 5 | yes | 10 | 278 | <0.35 | 92.2 |
| RTT99 | 19 | c.763C>T | p.R255X | Nonsense | TRD | severe | 9 | yes | 28 | 209 | <0.35 | 91.4 |
| RTT100 | 6 | c.808C>T | p.R270X | Nonsense | TRD-NLS | intermediate | 8 | no | 7 | 58 | <0.35 | 76.2 |
| RTT101 | 23 | c.455C>G | p.P152R | Missense | MBD | intermediate | 8 | yes | 31 | 250 | 6.91 | 191.2 |
| RTT111 | 17 | c.763C>T | p.R255X | Nonsense | TRD | intermediate | 7 | yes | 26 | 143 | <0.35 | 28.3 |
| RTT112 | 14 | c.808C>T | p.R270X | Nonsense | TRD-NLS | intermediate | 8 | yes | 25 | 226 | <0.35 | 73.9 |
| RTT114 | 15 | c.880C>T | p.R294X | Nonsense | TRD | intermediate | 6 | yes | 10 | 184 | <0.35 | 26.6 |
| RTT116 | 11 | c.808C>T | p.R270X | Nonsense | TRD-NLS | severe | 9 | yes | 10 | 233 | <0.35 | 45.7 |
| RTT119 | 2 | c.808C>T | p.R270X | Nonsense | TRD-NLS | severe | 9 | no | 74 | 96 | 2.54 | 71.2 |
| RTT123 | 22 | c.808C>T | p.R270X | Nonsense | TRD-NLS | severe | 11 | yes | 29 | 146 | 0.44 | 206.4 |
| RTT129 | 4 | c.502C>T | p.R168X | Nonsense | Inter-domain region | intermediate | 6 | yes | 14 | 111 | <0.35 | 41.5 |
| RTT132 | 8 | deletion exon 3. 4a. 4b | deletion exon 3. 4a. 4b | Large deletion | Large deletion | intermediate | 8 | yes | 38 | 231 | 1 | 55.5 |
| RTT134 | 11 | deletion exon 1. 2 | deletion exon 1. 2 | Large deletion | Large deletion | severe | 10 | yes | 22 | 288 | <0.35 | 32.2 |
| RTT135 | 18 | c.1152_1192del41 | p.P385fs | Frameshift in/del | C-term | intermediate | 6 | no | 31 | 209 | 2.01 | 143.4 |
| RTT136 | 9 | c.1157_1200del44 | p.L386fs | Frameshift in/del | C-term | intermediate | 8 | yes | 11 | 135 | <0.35 | 57.5 |
| RTT137 | 16 | c.[1111_1115del; 1116_1137inv] | p.H371LfsX34 | Frameshift in/del | C-term | severe | 10 | no | 37 | 172 | <0.35 | 122.3 |
| RTT138 | 2 | c.473C>T | p.T158M | Missense | MBD | severe | 11 | yes | 2 | 96 | <0.35 | 41.4 |
| RTT140 | 11 | c.398G>T | p.R133L | Missense | MBD | severe | 9 | yes | 9 | 160 | <0.35 | 48.2 |
| RTT142 | 10 | CDKL5 mutation | CDKL5 mutation | CDKL5 mutation | NA | intermediate | 8 | no | 24 | 135 | <0.35 | 500 |
| RTT147 | 2 | c.431delA | p.K144fs | Frameshift in/del | MBD | intermediate | 7 | no | NA | 130 | <0.35 | 56.8 |
| RTT151 | 6 | c.763C>T | p.R255X | Nonsense | TRD | intermediate | 6 | yes | 5 | 154 | 0.46 | 96.2 |
| RTT154 | 6 | c.905C>T | p.P302L | Missense | TRD | severe | 11 | no | 25 | 110 | 1 | 240.9 |
| RTT155 | 25 | CDKL5 mutation | CDKL5 mutation | CDKL5 mutation | NA | severe | 9 | yes | 36 | 283 | <0.35 | 124.4 |
| RTT158 | 6 | c.1164_1172del14 | p.P388fs | Frameshift in/del | C-term | intermediate | 6 | yes | 9 | 157 | <0.35 | 49.3 |
| RTT159 | 7 | c.502C>T | p.R168X | Nonsense | Inter-domain region | severe | 10 | yes | NA | 191 | <0.35 | 62.6 |
| RTT163 | 10 | c.808C>T | p.R270X | Nonsense | TRD-NLS | intermediate | 6 | no | 8 | 134 | <0.35 | NA |
| RTT164 | 15 | c.808C>T | p.R270X | Nonsense | TRD-NLS | severe | 11 | yes | 15 | 180 | <0.35 | NA |
| RTT165 | 15 | c.1157_1197del41 | p.L386fs | Frameshift in/del | C-term | intermediate | 7 | no | NA | 118 | NA | 31.9 |
| RTT167 | 8 | c.455C>G | p.P152R | Missense | MBD | severe | 10 | yes | 27 | 166 | <0.35 | 121.1 |
| RTT171 | 6 | c.916C>T | p.R306C | Missense | TRD | intermediate | 7 | yes | 19 | 111 | <0.35 | 59.3 |
| RTT172 | 13 | c.431delA | p.K144fs | Frameshift in/del | MBD | severe | 11 | yes | 21 | 182 | <0.35 | 19.7 |
| RTT177 | 11 | c.1162_1179del18 | p.P388_P393del | In-frame in/del | C-term | intermediate | 5 | NA | 7 | 140 | <0.35 | 61.5 |
| RTT190 | 14 | c.1165_1233del69ins21 | p.P389_P411del23ins7 | In-frame in/del | C-term | severe | 11 | yes | 49 | 427 | 0.75 | 206.6 |
| RTT199 | 2 | c.808C>T | p.R270X | Nonsense | TRD-NLS | intermediate | 8 | yes | NA | 40 | NA | 213.8 |
| RTT200 | 26 | c.1157_1197del41 | p.L386fs | Frameshift in/del | C-term | mild | 3 | yes | 62 | 273 | 1.51 | 204.3 |
| RTT215 | 19 | c.547G>C | p.G183R | Missense | Inter-domain region | intermediate | 5 | NA | NA | NA | NA | 15 |
| RTT250 | 22 | Large deletion | Large deletion | Large deletion | Large deletion | severe | 11 | yes | 109 | 210 | <0.35 | 29.8 |

*****According to RettBASE (http://mecp2.chw.edu.au/). CSS, Clinical Severity Score; ESR, Erythrocyte Sedimentation Rate; CRP, C-reactive protein; NA, not applicable. ^#^Clinic phenotype attributed according to the CSS (mild, 1-4; intermediate, 5-8; severe, 9-13).
