# Supplementary figures and images for: Altered gut microbiota in Rett syndrome
Source: Microbiome. 2016 Jul 30;4:41. doi: 10.1186/s40168-016-0185-y (PMC4967335; doi:10.1186/s40168-016-0185-y)

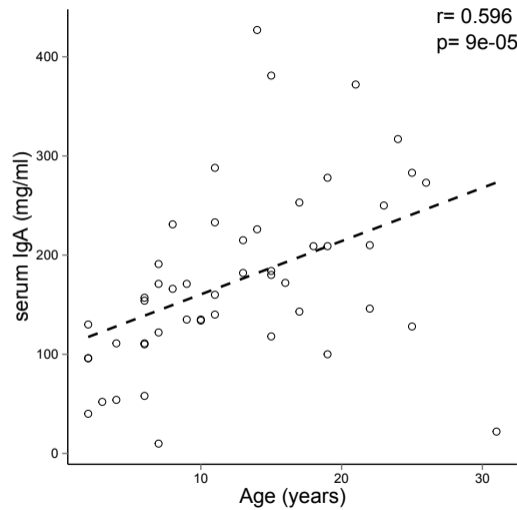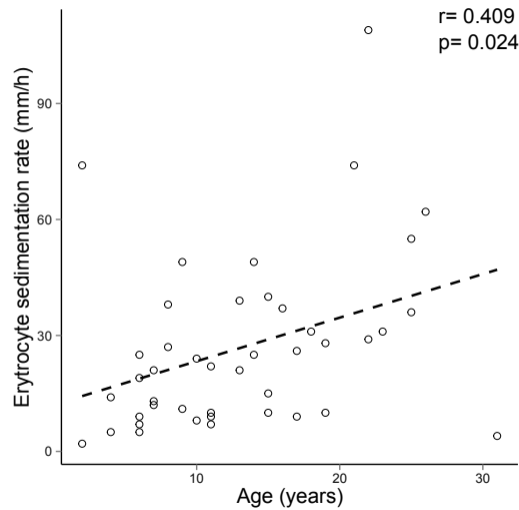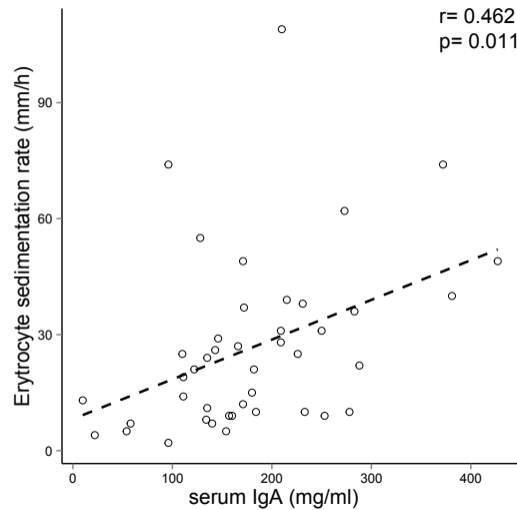

Supplement: Additional file 2: Figure S1. — Correlation plots of clinical data from RTT subjects. Significant positive correlations were observed among age, IgA and ESR. (PDF 37 kb) [file 40168_2016_185_MOESM2_ESM.pdf]

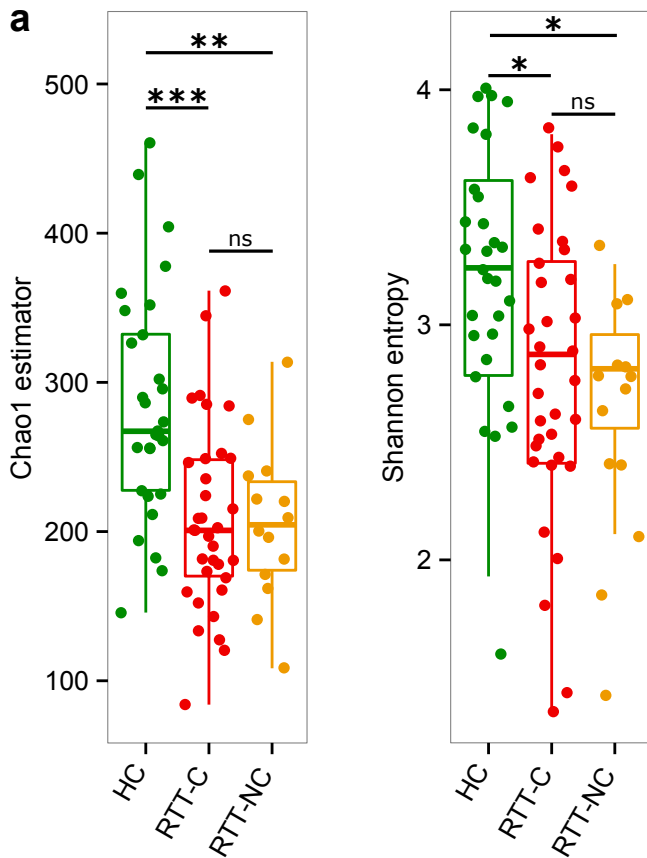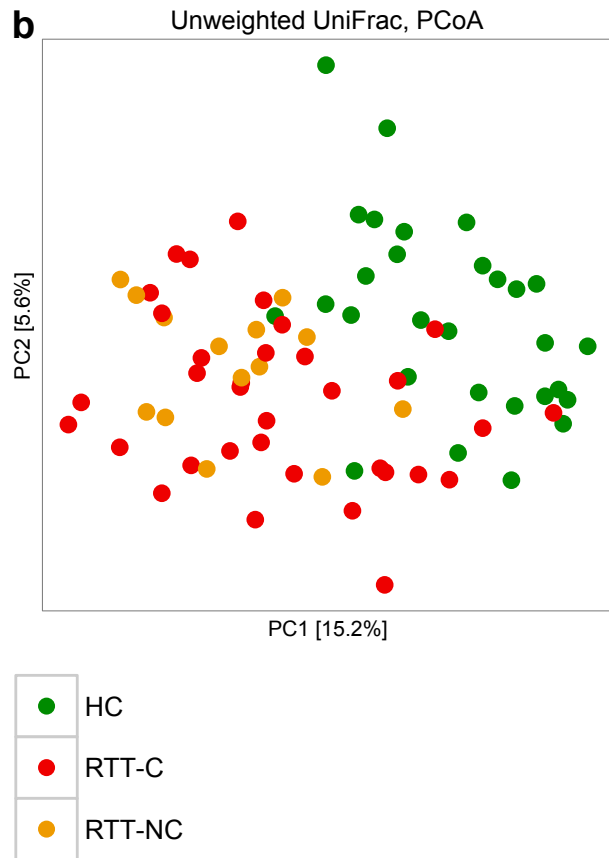

Supplement: Additional file 4: Figure S2. — Measures of bacterial diversity. a) Alpha-diversity estimated on the Chao1 estimator and the Shannon entropy; ***, p < 0.001; **, p < 0.01; *, p < 0.05; Wilcoxon rank-sum test. b) PCoA plot based on the unweighted UniFrac distance among samples analysed according to individuals’ health status. Constipated Rett syndrome subjects (RTT-C), non-constipated Rett syndrome subjects (RTT-NC) and healthy controls (HC) are coloured in red, orange or green, respectively. (PDF 58 kb) [file 40168_2016_185_MOESM4_ESM.pdf]

**a**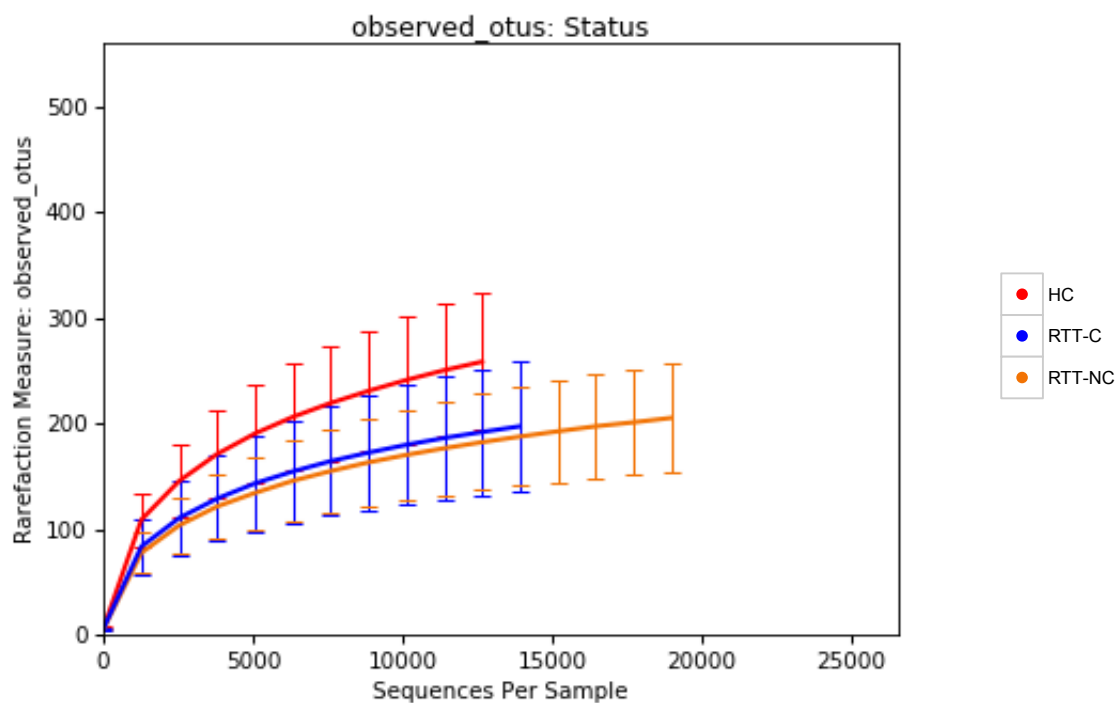**b**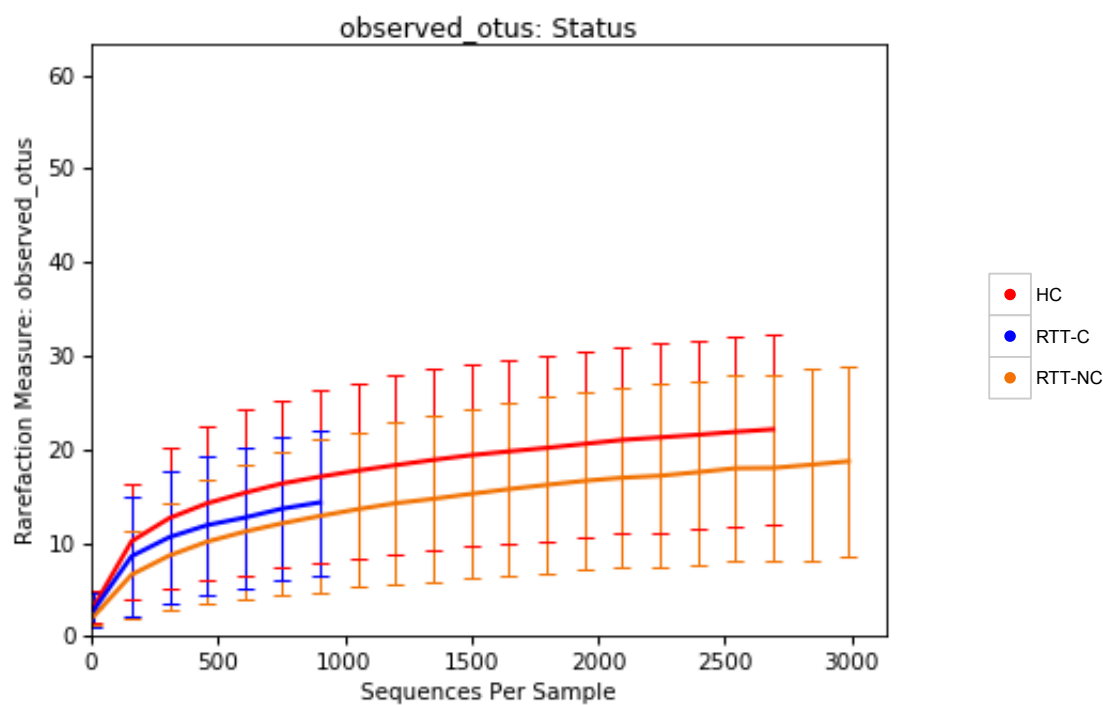

Supplement: Additional file 5: Figure S3. — Alpha-diversity rarefaction curves. The plot shows the alpha-diversity for HC, RTT-C and RTT-NC averaged over 100 independent rarefactions as a function of the rarefaction depth for a) the bacterial gut microbiota and b) the fungal gut microbiota. The points in the curves are the averages, while the whiskers represent the standard deviations. (PDF 47 kb) [file 40168_2016_185_MOESM5_ESM.pdf]

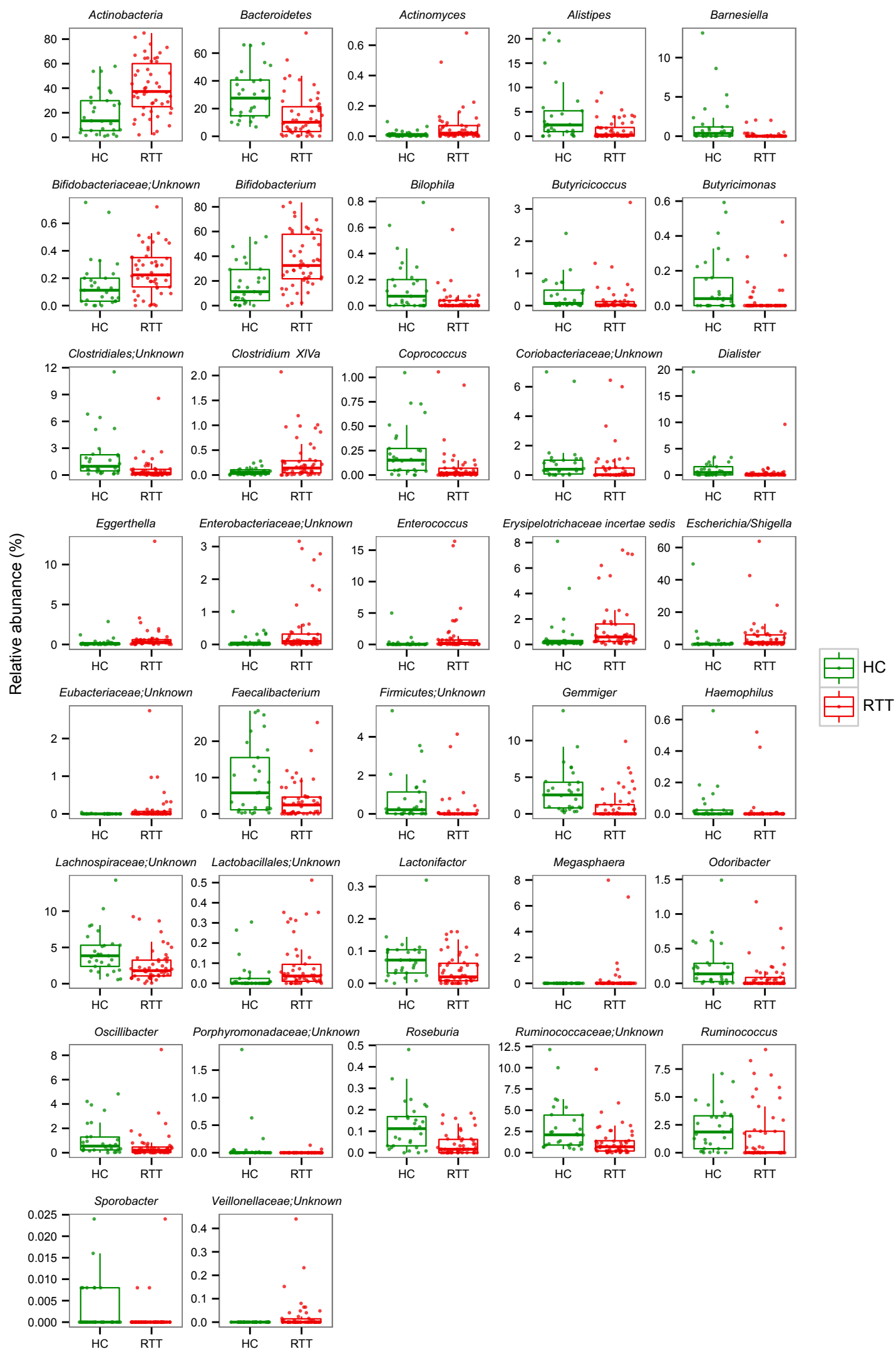

Supplement: Additional file 11: Figure S7. — Bacterial taxa which relative abundances were significantly different (p < 0.05; Wilcoxon rank-sum test) between healthy controls (HC) and Rett syndrome (RTT) subjects. (PDF 319 kb) [file 40168_2016_185_MOESM11_ESM.pdf]

RTT      HC

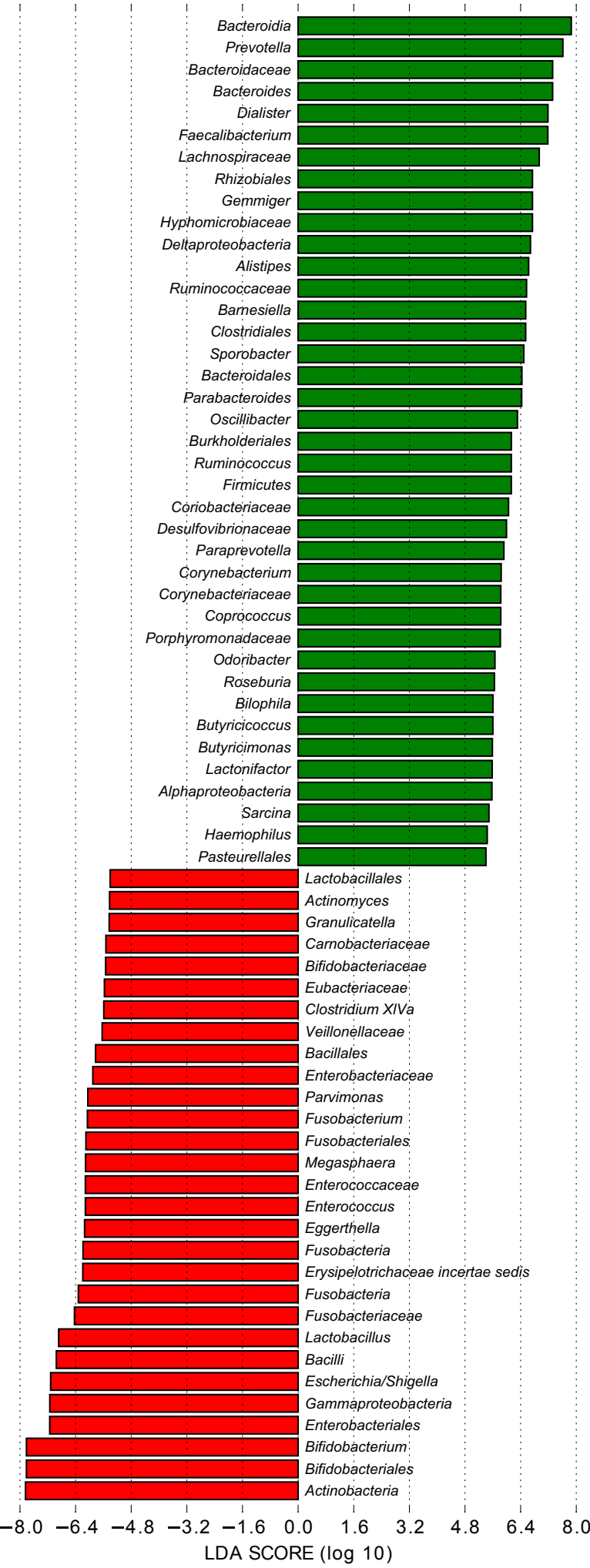

Supplement: Additional file 12: Figure S8. — LDA scores of the most discriminant bacterial taxa identified by LEfSe. Positive and negative LDA scores indicate the taxa enriched in healthy controls (HC) and Rett syndrome (RTT) subjects, respectively. (PDF 62 kb) [file 40168_2016_185_MOESM12_ESM.pdf]

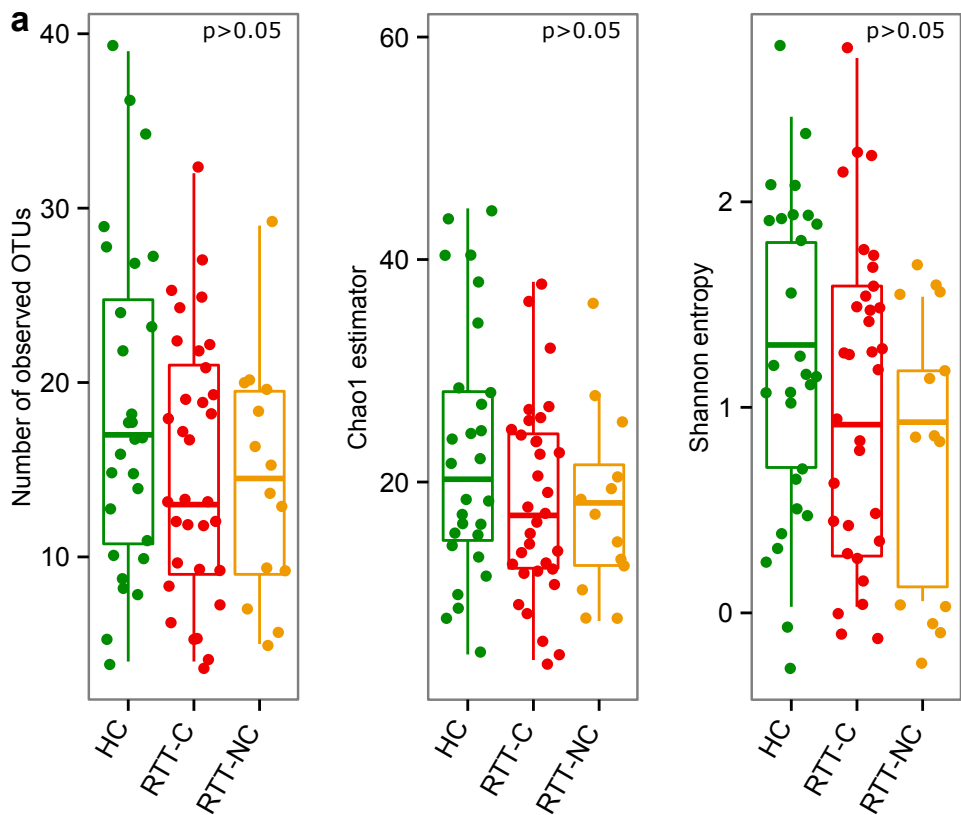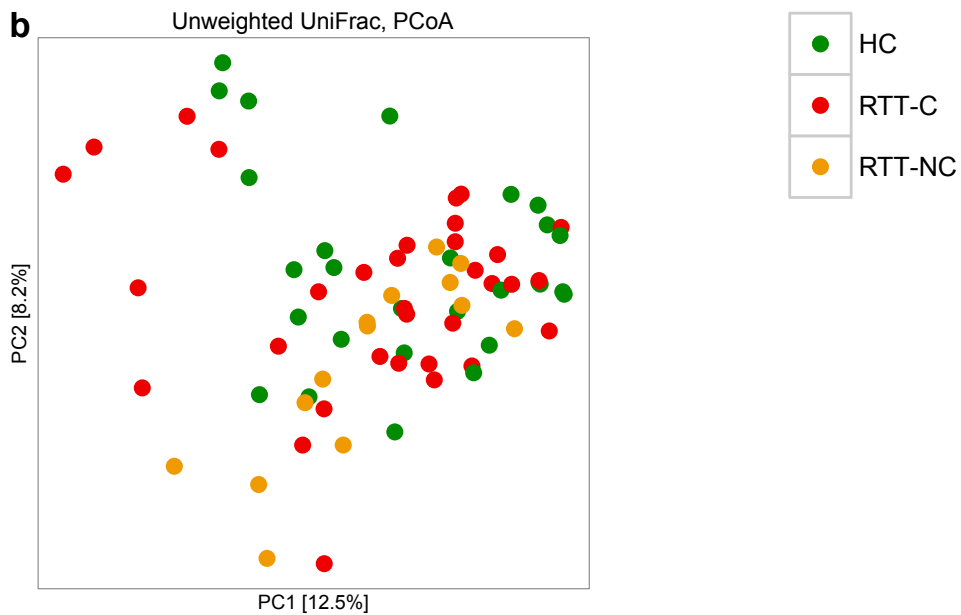

Supplement: Additional file 14: Figure S9. — Measures of fungal diversity. a) Three estimators of alpha-diversity have been calculated: the number of observed OTUs, the Chao1 estimator and the Shannon entropy; b) PCoA plot based on the unweighted UniFrac distance among samples analysed according to individuals’ health status. Constipated Rett syndrome subjects (RTT-C), non-constipated Rett syndrome subjects (RTT-NC) and healthy controls (HC) are coloured in red, orange or green, respectively. (PDF 63 kb) [file 40168_2016_185_MOESM14_ESM.pdf]

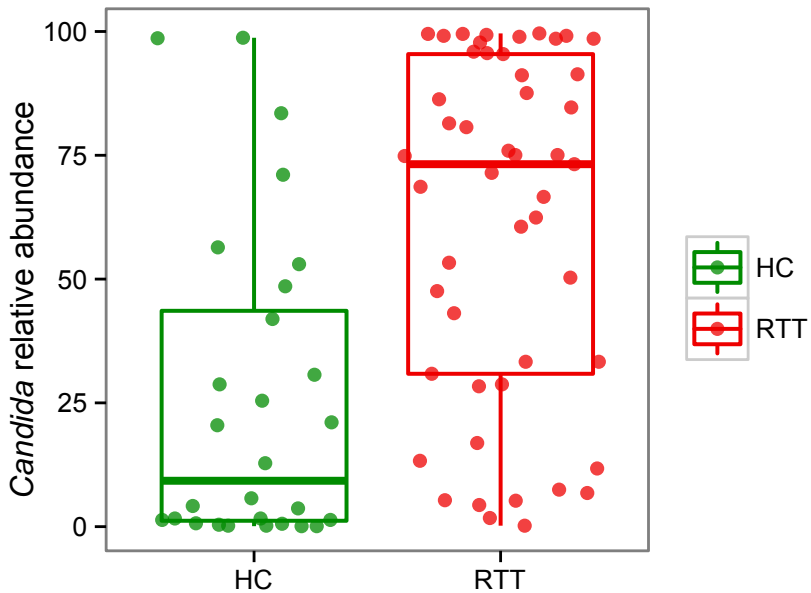

Supplement: Additional file 16: Figure S11. — Candida relative abundance in the gut microbiota of healthy controls (HC) and Rett syndrome (RTT) subjects (p < 0.05; Wilcoxon rank-sum test). (PDF 20 kb) [file 40168_2016_185_MOESM16_ESM.pdf]

**a**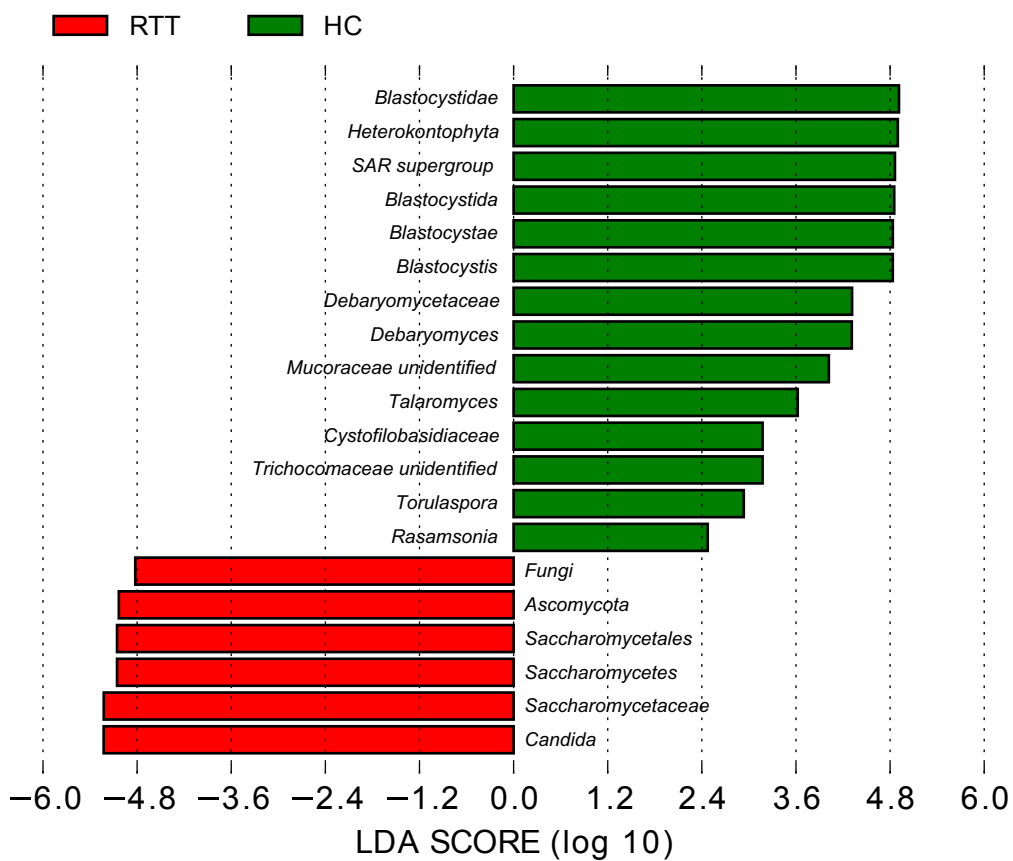**b**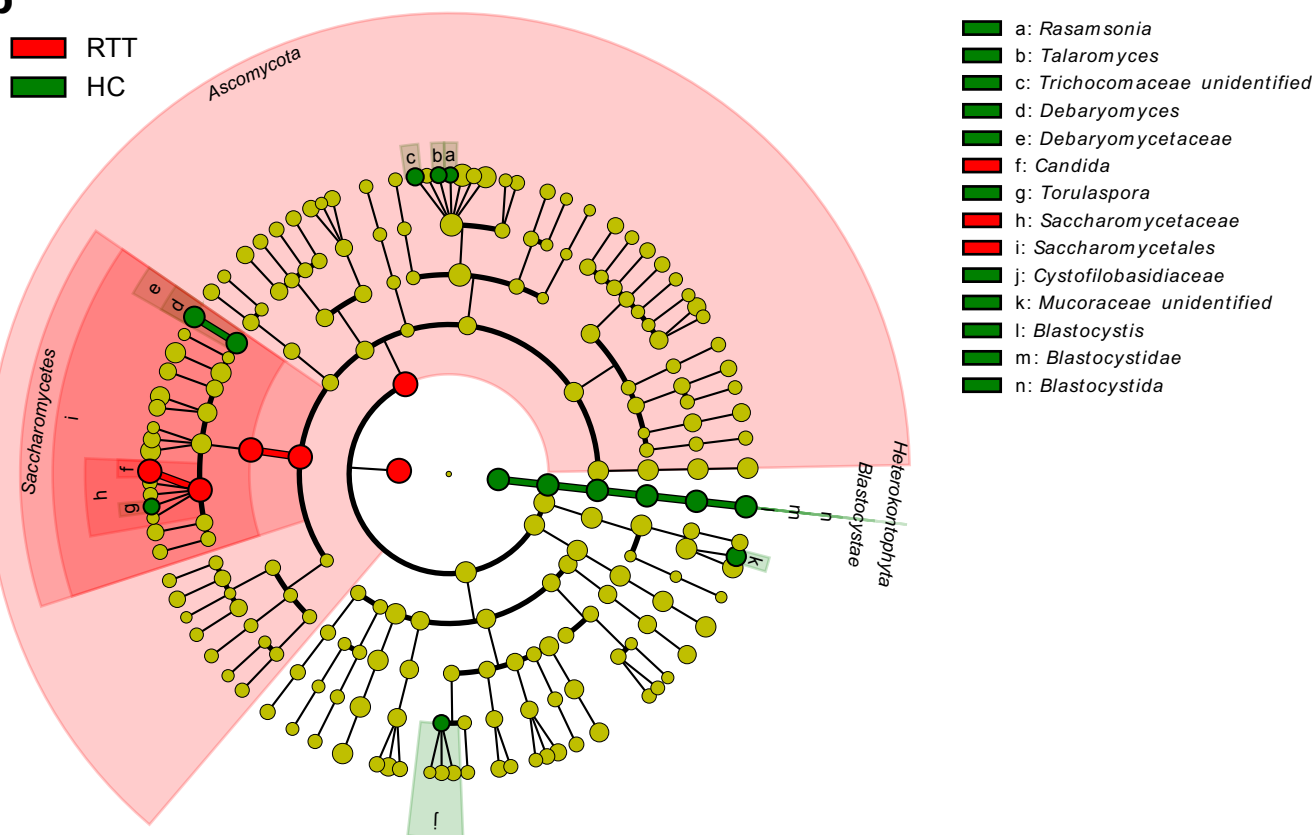

Supplement: Additional file 17: Figure S12. — a) LDA scores of the most discriminant fungal taxa identified by LEfSe. Positive and negative LDA scores indicate the taxa enriched in healthy controls (HC) and Rett syndrome (RTT) subjects, respectively. b) Cladogram showing the most discriminative fungal clades identified by LEfSe. Coloured regions/branches indicate differences in the fungal population structure between Rett syndrome (RTT) subjects and healthy controls (HC). Regions in red indicate clades that were enriched in RTT subjects compared to those in HC, while regions in green indicate clades that were enriched in HC compared to those in RTT subjects. (PDF 116 kb) [file 40168_2016_185_MOESM17_ESM.pdf]
