# Supplementary material for: Altered gut microbiota in Rett syndrome
Source: Microbiome. 2016 Jul 30;4:41. doi: 10.1186/s40168-016-0185-y (PMC4967335; doi:10.1186/s40168-016-0185-y)
Supplement: Additional file 3: Table S2. — Spearman’s correlation analysis among RTT clinical data. (DOCX 14 kb) [file 40168_2016_185_MOESM3_ESM.docx]

| *Spearman's r* | Age | CSS | Constipation | IgA | ESR | Calprotectin |
| --- | --- | --- | --- | --- | --- | --- |
| Age | 1.000 | 0.090 | 0.350 | 0.596 | 0.409 | 0.177 |
|  | **CSS** | 1.000 | 0.137 | 0.211 | 0.234 | 0.092 |
|  |  | **Constipation** | 1.000 | 0.430 | 0.051 | -0.089 |
|  |  |  | **IgA** | 1.000 | 0.462 | 0.017 |
|  |  |  |  | **ESR** | 1.000 | 0.205 |
|  |  |  |  |  | **Calprotectin** | 1.000 |
|  |  |  |  |  |  |  |
|  |  |  |  |  |  |  |
| *p-value* | **Age** | **CSS** | **Constipation** | **IgA** | **ESR** | **Calprotectin** |
| Age | 0.000 | 0.642 | 0.045 | 0.00009 | 0.024 | 0.379 |
|  | **CSS** | 0.000 | 0.539 | 0.323 | 0.323 | 0.642 |
|  |  | **Constipation** | 0.000 | 0.011 | 0.800 | 0.642 |
|  |  |  | **IgA** | 0.000 | 0.011 | 0.907 |
|  |  |  |  | **ESR** | 0.000 | 0.374 |
|  |  |  |  |  | **Calprotectin** | 0.000 |

**Supplementary Table 2:** Spearman’s correlation analysis among RTT clinical data.
