# Supplementary material for: Altered gut microbiota in Rett syndrome
Source: Microbiome. 2016 Jul 30;4:41. doi: 10.1186/s40168-016-0185-y (PMC4967335; doi:10.1186/s40168-016-0185-y)
Supplement: Additional file 6: Figure S4. — Multiple-rarefaction PCoA plots. Each PCoA replicate was optimally superimposed by Procrustes analysis on the master PCoA scatter plot (used in the main text). Points represent the average location of 100 rarefaction replicates. Ellipses show the 95 % confidence region assuming a multivariate normal distribution. a) PCoA plots of bacterial beta-diversity based on the unweighted and weighted UniFrac distances and the Bray-Curtis dissimilarity analysed according to individuals’ health status; b) PCoA plots of fungal beta-diversity based on the unweighted and weighted UniFrac distances and the Bray-Curtis dissimilarity analysed according to individuals’ health status. Constipated Rett syndrome subjects (RTT-C), non-constipated Rett syndrome subjects (RTT-NC) and healthy controls (HC) are coloured in red, orange or green, respectively. (PDF 185 kb) [file 40168_2016_185_MOESM6_ESM.pdf]

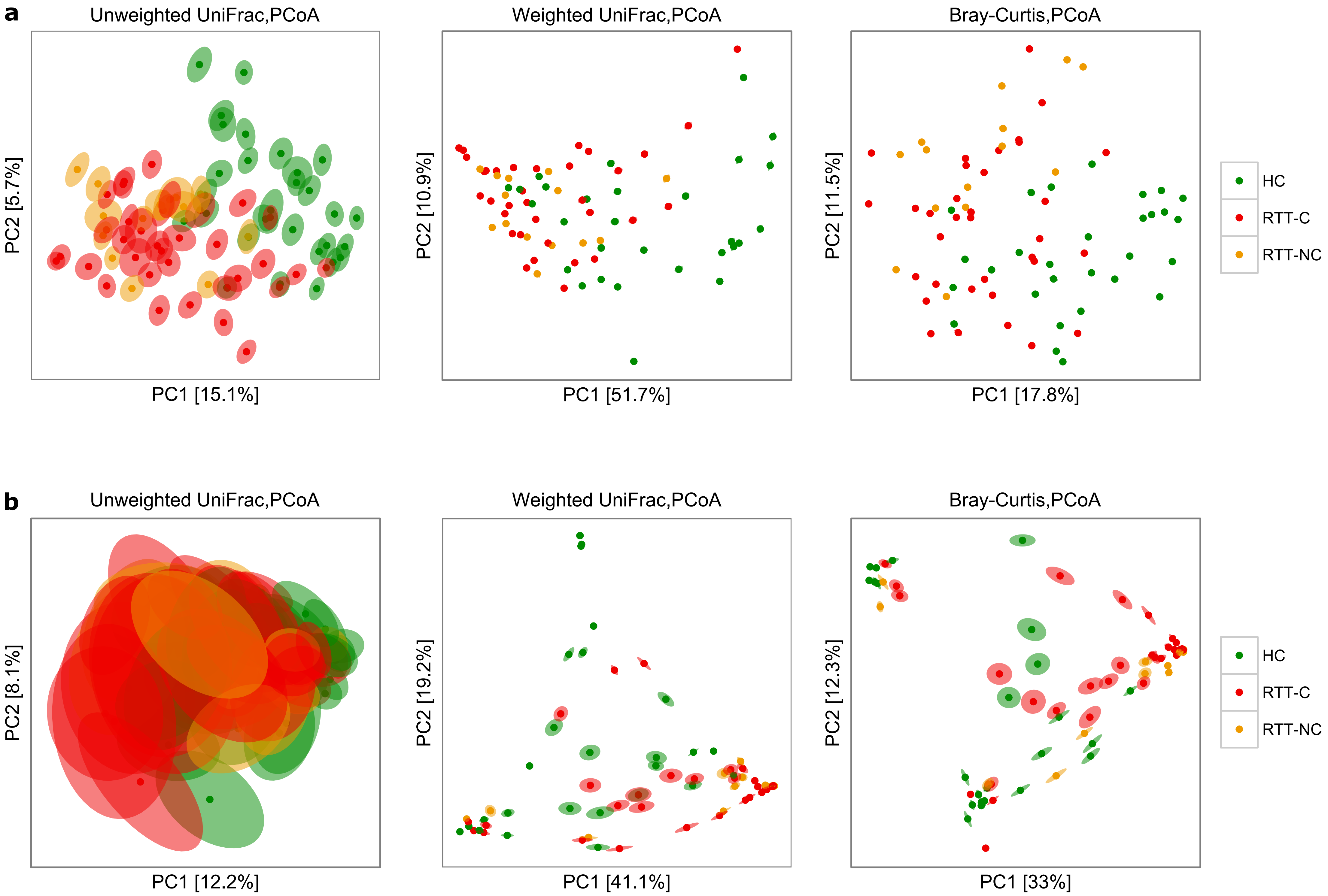
a
Unweighted UniFrac,PCoA
PC2 [5.7%]
PC1 [15.1%]
Weighted UniFrac,PCoA
PC2 [10.9%]
PC1 [51.7%]
Bray-Curtis,PCoA
PC2 [11.5%]
PC1 [17.8%]
HC
RTT-C
RTT-NC
b
Unweighted UniFrac,PCoA
PC2 [8.1%]
PC1 [12.2%]
Weighted UniFrac,PCoA
PC2 [19.2%]
PC1 [41.1%]
Bray-Curtis,PCoA
PC2 [12.3%]
PC1 [33%]
HC
RTT-C
RTT-NC
