# Supplementary material for: Altered gut microbiota in Rett syndrome
Source: Microbiome. 2016 Jul 30;4:41. doi: 10.1186/s40168-016-0185-y (PMC4967335; doi:10.1186/s40168-016-0185-y)
Supplement: Additional file 7: Figure S5. — PERMANOVA p values distribution. For each rarefaction replicate (n = 100), a PERMANOVA test was conducted to assess the robustness of the results over the rarefaction. a) PERMANOVA p values distributions of bacterial beta-diversity based on the unweighted and weighted UniFrac distances and the Bray-Curtis dissimilarity analysed according to individuals’ health status; b) PERMANOVA p values distributions of fungal beta-diversity based on the unweighted and weighted UniFrac distances and the Bray-Curtis dissimilarity analysed according to individuals’ health status. (PDF 27 kb) [file 40168_2016_185_MOESM7_ESM.pdf]

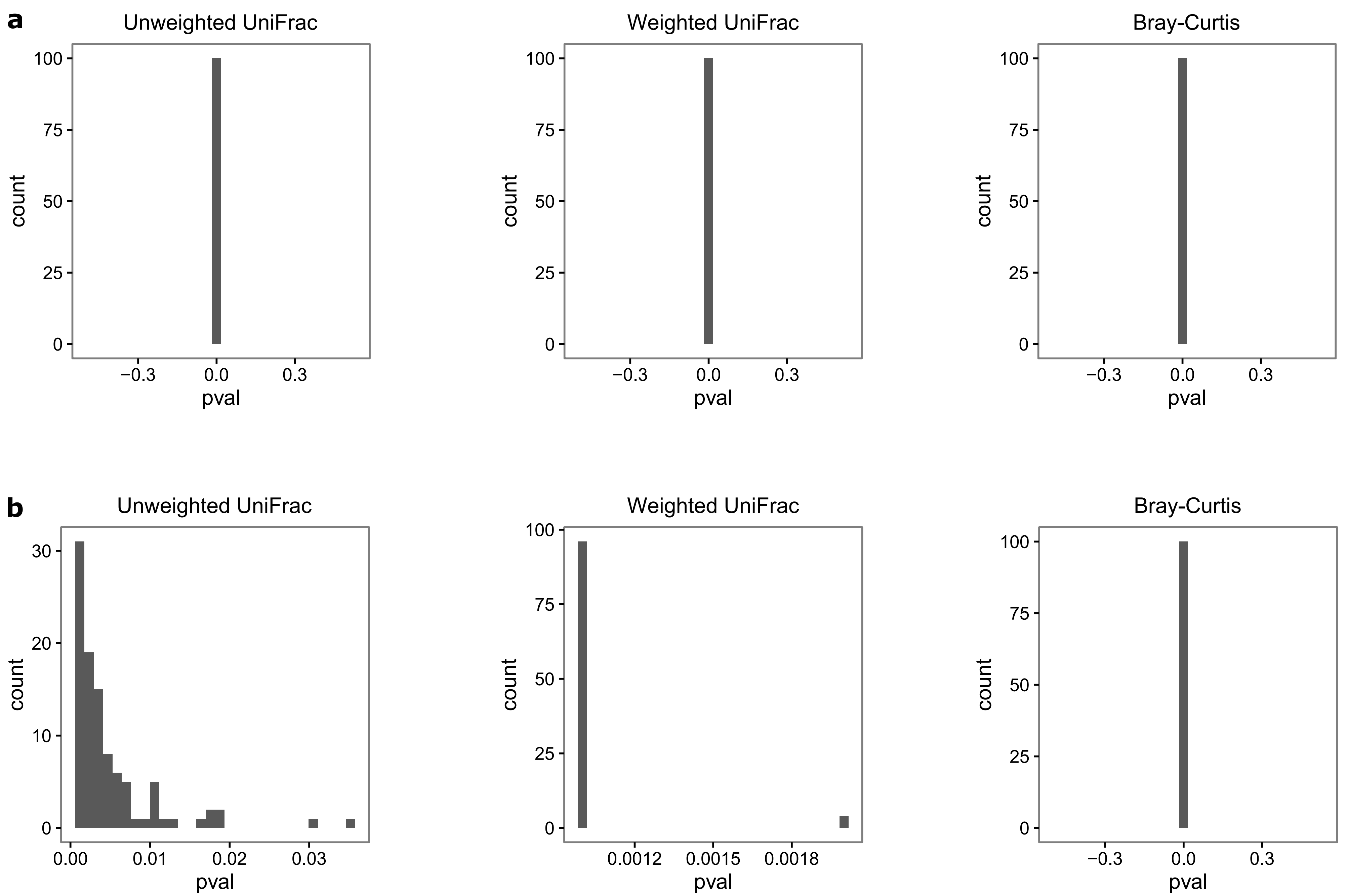
a
Unweighted UniFrac
Weighted UniFrac
Bray-Curtis
count
pval
100
75
50
25
0
−0.3
0.0
0.3
b
Unweighted UniFrac
Weighted UniFrac
Bray-Curtis
30
20
10
0.00
0.01
0.02
0.03
0.0012
0.0015
0.0018
