# Supplementary material for: Altered gut microbiota in Rett syndrome
Source: Microbiome. 2016 Jul 30;4:41. doi: 10.1186/s40168-016-0185-y (PMC4967335; doi:10.1186/s40168-016-0185-y)
Supplement: Additional file 8: Table S3. — Mean relative abundance (%) ± standard deviation (SD) of bacterial OTUs at the phylum and genus levels in Rett syndrome (RTT) subjects and healthy controls (HC). (DOCX 32 kb) [file 40168_2016_185_MOESM8_ESM.docx]

**Supplementary Table 3:** Mean relative abundance (%) ± standard deviation (SD) of bacterial OTUs at phylum and genus levels in Rett syndrome (RTT) subjects and healthy controls (HC).

|  | RTT | | HC | |
| --- | --- | --- | --- | --- |
| *Phylum* | **mean** | **SD** | **mean** | **SD** |
| *Firmicutes* | 36.6560 | 16.3355 | 43.6145 | 14.4643 |
| *Actinobacteria* | 40.5576 | 21.7507 | 19.5674 | 17.4821 |
| *Bacteroidetes* | 15.1573 | 15.9453 | 30.2396 | 17.9768 |
| *Proteobacteria* | 7.0502 | 11.3086 | 6.2200 | 9.7279 |
| *Unknown* | 0.4947 | 0.8526 | 0.3044 | 0.2567 |
| *Verrucomicrobia* | 0.0589 | 0.1550 | 0.0450 | 0.0804 |
| *Synergistetes* | 0.0110 | 0.0591 | 0.0028 | 0.0120 |
| *Fusobacteria* | 0.0080 | 0.0210 | 0.0003 | 0.0015 |
| *TM7* | 0.0027 | 0.0064 | 0.0019 | 0.0104 |
| *Lentisphaerae* | 0.0022 | 0.0158 | 0.0022 | 0.0091 |
| *Elusimicrobia* | 0.0000 | 0.0000 | 0.0017 | 0.0089 |
| *Cyanobacteria* | 0.0013 | 0.0061 | 0.0003 | 0.0015 |

|  | RTT | | HC | |
| --- | --- | --- | --- | --- |
| *Genus* | **mean** | **SD** | **mean** | **SD** |
| *Bifidobacterium* | 36.7042 | 21.9180 | 17.2935 | 16.4407 |
| *Bacteroides* | 12.3152 | 13.8314 | 18.3145 | 15.2315 |
| *Faecalibacterium* | 3.6678 | 4.9853 | 9.2303 | 9.4653 |
| *Lachnospiracea incertae sedis* | 4.6103 | 4.9332 | 3.9995 | 2.5715 |
| *Blautia* | 4.7655 | 7.0012 | 3.7541 | 5.6668 |
| *Escherichia/Shigella* | 5.2332 | 11.0378 | 2.4657 | 9.0906 |
| *Lachnospiraceae;Unknown* | 2.5140 | 2.2392 | 4.3575 | 2.9834 |
| *Alistipes* | 1.3665 | 2.0459 | 4.7119 | 6.2269 |
| *Streptococcus* | 2.3251 | 4.1986 | 2.9697 | 11.3418 |
| *Gemmiger* | 1.1117 | 2.0365 | 3.1772 | 3.1064 |
| *Ruminococcaceae;Unknown* | 1.2905 | 1.7899 | 2.9735 | 2.8355 |
| *Ruminococcus* | 1.5287 | 2.5080 | 2.2076 | 1.9194 |
| *Clostridium XI* | 1.5268 | 1.8281 | 1.2691 | 1.3289 |
| *Collinsella* | 1.9867 | 3.3628 | 0.7221 | 1.0007 |
| *Clostridiales;Unknown* | 0.6234 | 1.3096 | 2.0696 | 2.5707 |
| *Parabacteroides* | 1.0523 | 1.9446 | 1.4590 | 1.5945 |
| *Clostridium XVIII* | 1.3167 | 1.8063 | 1.1703 | 1.4937 |
| *Anaerostipes* | 1.2669 | 1.5410 | 1.2065 | 1.0610 |
| *Prevotella* | 0.0285 | 0.0968 | 2.2137 | 10.4531 |
| *Erysipelotrichaceae incertae sedis* | 1.3897 | 2.0083 | 0.7190 | 1.6414 |
| *Dialister* | 0.3793 | 1.3711 | 1.5186 | 3.5555 |
| *Barnesiella* | 0.1787 | 0.4704 | 1.5752 | 2.8878 |
| *Oscillibacter* | 0.5675 | 1.3085 | 1.1060 | 1.3587 |
| *Enterococcus* | 1.2751 | 3.2763 | 0.2677 | 0.9199 |
| *Coriobacteriaceae;Unknown* | 0.5619 | 1.3252 | 0.9567 | 1.6284 |
| *Lactobacillus* | 1.2687 | 3.8816 | 0.0447 | 0.0689 |
| *Clostridium sensu stricto* | 0.6903 | 1.5974 | 0.6045 | 0.7939 |
| *Prevotellaceae;Unknown* | 0.0507 | 0.1724 | 1.1107 | 3.8195 |
| *Firmicutes;Unknown* | 0.2289 | 0.7742 | 0.7980 | 1.2740 |
| *Eggerthella* | 0.7638 | 1.8753 | 0.2498 | 0.5586 |
| *Clostridium IV* | 0.4129 | 0.6337 | 0.4747 | 0.5825 |
| *Veillonella* | 0.8229 | 2.1017 | 0.0547 | 0.1136 |
| *Unknown* | 0.4947 | 0.8526 | 0.3044 | 0.2522 |
| *Megamonas* | 0.6157 | 2.7667 | 0.0803 | 0.4250 |
| *Phascolarctobacterium* | 0.1319 | 0.2905 | 0.4954 | 0.8446 |
| *Flavonifractor* | 0.2888 | 0.3677 | 0.2713 | 0.3345 |
| *Enterobacteriaceae;Unknown* | 0.4359 | 0.8197 | 0.1041 | 0.2101 |
| *Butyricicoccus* | 0.2009 | 0.5159 | 0.3265 | 0.4766 |
| *Catenibacterium* | 0.4675 | 2.6156 | 0.0276 | 0.1461 |
| *Bifidobacteriaceae;Unknown* | 0.2497 | 0.1642 | 0.1615 | 0.1800 |
| *Dorea* | 0.1787 | 0.2538 | 0.2219 | 0.2247 |
| *Megasphaera* | 0.3791 | 1.4683 | 0.0000 | 0.0000 |
| *Clostridium XlVa* | 0.2955 | 0.4094 | 0.0704 | 0.0715 |
| *Odoribacter* | 0.1001 | 0.2170 | 0.2448 | 0.3198 |
| *Coprococcus* | 0.0842 | 0.1999 | 0.2357 | 0.2602 |
| *Acidaminococcus* | 0.1823 | 0.5856 | 0.1223 | 0.4516 |
| *Turicibacter* | 0.2044 | 0.6219 | 0.0696 | 0.1336 |
| *Clostridium XlVb* | 0.1561 | 0.2811 | 0.1063 | 0.1835 |
| *Bacteroidetes;Unknown* | 0.0050 | 0.0194 | 0.2186 | 0.7603 |
| *Olsenella* | 0.1324 | 0.6047 | 0.0618 | 0.2847 |
| *Eubacterium* | 0.0751 | 0.1190 | 0.1143 | 0.1453 |
| *Bilophila* | 0.0355 | 0.0899 | 0.1460 | 0.1932 |
| *Paraprevotella* | 0.0125 | 0.0557 | 0.1568 | 0.3086 |
| *Roseburia* | 0.0403 | 0.0522 | 0.1231 | 0.1111 |
| *Parasutterella* | 0.0953 | 0.3672 | 0.0588 | 0.1199 |
| *Butyricimonas* | 0.0331 | 0.0896 | 0.1140 | 0.1654 |
| *Eubacteriaceae;Unknown* | 0.1372 | 0.4310 | 0.0019 | 0.0078 |
| *Desulfovibrio* | 0.0482 | 0.1399 | 0.0789 | 0.1998 |
| *Lactobacillales;Unknown* | 0.0874 | 0.1231 | 0.0334 | 0.0749 |
| *Lactococcus* | 0.1087 | 0.4339 | 0.0097 | 0.0279 |
| *Lactonifactor* | 0.0413 | 0.0465 | 0.0734 | 0.0600 |
| *Porphyromonadaceae;Unknown* | 0.0040 | 0.0211 | 0.1021 | 0.3562 |
| *Akkermansia* | 0.0584 | 0.1546 | 0.0450 | 0.0790 |
| *Sarcina* | 0.0018 | 0.0075 | 0.0961 | 0.3427 |
| *Granulicatella* | 0.0652 | 0.1346 | 0.0315 | 0.1098 |
| *Peptostreptococcaceae;Unknown* | 0.0551 | 0.0655 | 0.0386 | 0.0532 |
| *Coprobacillus* | 0.0453 | 0.1166 | 0.0362 | 0.0851 |
| *Actinomyces* | 0.0631 | 0.1206 | 0.0155 | 0.0213 |
| *Anaerotruncus* | 0.0519 | 0.1073 | 0.0248 | 0.0678 |
| *Clostridia;Unknown* | 0.0115 | 0.0386 | 0.0638 | 0.2839 |
| *Erysipelotrichaceae;Unknown* | 0.0499 | 0.1590 | 0.0229 | 0.0395 |
| *Haemophilus* | 0.0207 | 0.0938 | 0.0491 | 0.1256 |
| *Gordonibacter* | 0.0303 | 0.0561 | 0.0337 | 0.1103 |
| *Slackia* | 0.0171 | 0.0546 | 0.0425 | 0.1459 |
| *Clostridiaceae 1;Unknown* | 0.0157 | 0.0334 | 0.0389 | 0.0719 |
| *Mitsuokella* | 0.0311 | 0.2185 | 0.0213 | 0.1125 |
| *Sutterella* | 0.0154 | 0.0480 | 0.0356 | 0.1047 |
| *Holdemania* | 0.0239 | 0.0409 | 0.0232 | 0.0282 |
| *Alphaproteobacteria;Unknown* | 0.0019 | 0.0082 | 0.0439 | 0.1366 |
| *Proteobacteria;Unknown* | 0.0232 | 0.1641 | 0.0039 | 0.0165 |
| *Veillonellaceae;Unknown* | 0.0261 | 0.0729 | 0.0000 | 0.0000 |
| *Enterorhabdus* | 0.0080 | 0.0371 | 0.0177 | 0.0822 |
| *Burkholderiales;Unknown* | 0.0005 | 0.0034 | 0.0248 | 0.1123 |
| *Allisonella* | 0.0216 | 0.0912 | 0.0006 | 0.0029 |
| *Peptoniphilus* | 0.0118 | 0.0479 | 0.0066 | 0.0236 |
| *Varibaculum* | 0.0155 | 0.0898 | 0.0022 | 0.0103 |
| *Peptostreptococcus* | 0.0163 | 0.0536 | 0.0008 | 0.0024 |
| *Desulfovibrionaceae;Unknown* | 0.0002 | 0.0011 | 0.0132 | 0.0470 |
| *Pyramidobacter* | 0.0106 | 0.0590 | 0.0025 | 0.0117 |
| *Sutterellaceae;Unknown* | 0.0062 | 0.0305 | 0.0063 | 0.0270 |
| *Anaerofustis* | 0.0072 | 0.0131 | 0.0044 | 0.0112 |
| *Porphyromonas* | 0.0061 | 0.0353 | 0.0052 | 0.0187 |
| *Anaerococcus* | 0.0088 | 0.0231 | 0.0019 | 0.0058 |
| *Pediococcus* | 0.0093 | 0.0432 | 0.0011 | 0.0046 |
| *Morganella* | 0.0077 | 0.0236 | 0.0011 | 0.0058 |
| *Corynebacterium* | 0.0056 | 0.0100 | 0.0030 | 0.0118 |
| *Fusobacterium* | 0.0080 | 0.0210 | 0.0003 | 0.0015 |
| *Bacteroidales;Unknown* | 0.0011 | 0.0040 | 0.0069 | 0.0160 |
| *Clostridiales Incertae Sedis XI;Unknown* | 0.0027 | 0.0181 | 0.0052 | 0.0263 |
| *Parvimonas* | 0.0074 | 0.0259 | 0.0003 | 0.0015 |
| *Finegoldia* | 0.0056 | 0.0173 | 0.0019 | 0.0062 |
| *Weissella* | 0.0067 | 0.0453 | 0.0003 | 0.0015 |
| *Gemella* | 0.0050 | 0.0110 | 0.0014 | 0.0042 |
| *Actinomycetales;Unknown* | 0.0051 | 0.0135 | 0.0008 | 0.0032 |
| *Staphylococcus* | 0.0059 | 0.0198 | 0.0000 | 0.0000 |
| *Proteus* | 0.0058 | 0.0206 | 0.0000 | 0.0000 |
| *Oxalobacter* | 0.0011 | 0.0061 | 0.0044 | 0.0134 |
| *Anaerofilum* | 0.0029 | 0.0107 | 0.0025 | 0.0060 |
| *Pasteurellaceae;Unknown* | 0.0030 | 0.0136 | 0.0022 | 0.0091 |
| *TM7 genera incertae sedis* | 0.0027 | 0.0064 | 0.0019 | 0.0102 |
| *Peptococcus* | 0.0043 | 0.0153 | 0.0003 | 0.0015 |
| *Victivallis* | 0.0022 | 0.0158 | 0.0022 | 0.0089 |
| *Actinobacteria;Unknown* | 0.0011 | 0.0051 | 0.0033 | 0.0080 |
| *Howardella* | 0.0043 | 0.0306 | 0.0000 | 0.0000 |
| *Anaerovorax* | 0.0003 | 0.0023 | 0.0039 | 0.0148 |
| *Rikenella* | 0.0011 | 0.0079 | 0.0030 | 0.0161 |
| *Sporobacter* | 0.0008 | 0.0037 | 0.0030 | 0.0057 |
| *Anaeroglobus* | 0.0037 | 0.0186 | 0.0000 | 0.0000 |
| *Atopobium* | 0.0034 | 0.0111 | 0.0003 | 0.0015 |
| *Kocuria* | 0.0030 | 0.0113 | 0.0000 | 0.0000 |
| *Hydrogenoanaerobacterium* | 0.0000 | 0.0000 | 0.0028 | 0.0146 |
| *Dysgonomonas* | 0.0021 | 0.0136 | 0.0006 | 0.0029 |
| *Xylanibacter* | 0.0003 | 0.0023 | 0.0022 | 0.0078 |
| *Actinomycetaceae;Unknown* | 0.0021 | 0.0082 | 0.0003 | 0.0015 |
| *Propionibacterium* | 0.0018 | 0.0073 | 0.0006 | 0.0029 |
| *Solobacterium* | 0.0021 | 0.0094 | 0.0000 | 0.0000 |
| *Mogibacterium* | 0.0005 | 0.0034 | 0.0014 | 0.0052 |
| *Deltaproteobacteria;Unknown* | 0.0002 | 0.0011 | 0.0017 | 0.0049 |
| *Elusimicrobium* | 0.0000 | 0.0000 | 0.0017 | 0.0088 |
| *Succiniclasticum* | 0.0016 | 0.0113 | 0.0000 | 0.0000 |
| *Streptophyta* | 0.0013 | 0.0061 | 0.0003 | 0.0015 |
| *Desulfovibrionales;Unknown* | 0.0006 | 0.0027 | 0.0008 | 0.0044 |
| *Comamonas* | 0.0011 | 0.0079 | 0.0003 | 0.0015 |
| *Acinetobacter* | 0.0008 | 0.0046 | 0.0006 | 0.0020 |
| *Mobiluncus* | 0.0002 | 0.0011 | 0.0008 | 0.0032 |
| *Cardiobacterium* | 0.0000 | 0.0000 | 0.0008 | 0.0044 |
| *Scardovia* | 0.0008 | 0.0037 | 0.0000 | 0.0000 |
| *Pseudomonas* | 0.0008 | 0.0040 | 0.0000 | 0.0000 |
| *Cloacibacillus* | 0.0005 | 0.0034 | 0.0003 | 0.0015 |
| *Pseudoramibacter* | 0.0006 | 0.0036 | 0.0000 | 0.0000 |
| *Clostridiales Incertae Sedis XIII;Unknown* | 0.0006 | 0.0045 | 0.0000 | 0.0000 |
| *Rothia* | 0.0003 | 0.0023 | 0.0003 | 0.0015 |
| *Gordonia* | 0.0000 | 0.0000 | 0.0006 | 0.0029 |
| *Paraeggerthella* | 0.0000 | 0.0000 | 0.0006 | 0.0029 |
| *Actinobaculum* | 0.0005 | 0.0025 | 0.0000 | 0.0000 |
| *Trueperella* | 0.0005 | 0.0034 | 0.0000 | 0.0000 |
| *Abiotrophia* | 0.0005 | 0.0034 | 0.0000 | 0.0000 |
| *Murdochiella* | 0.0005 | 0.0025 | 0.0000 | 0.0000 |
| *Schwartzia* | 0.0005 | 0.0034 | 0.0000 | 0.0000 |
| *Rhizobacter* | 0.0005 | 0.0034 | 0.0000 | 0.0000 |
| *Puniceicoccaceae;Unknown* | 0.0005 | 0.0034 | 0.0000 | 0.0000 |
| *Leclercia* | 0.0002 | 0.0011 | 0.0003 | 0.0015 |
| *Arcanobacterium* | 0.0003 | 0.0023 | 0.0000 | 0.0000 |
| *Devosia* | 0.0003 | 0.0023 | 0.0000 | 0.0000 |
| *Enhydrobacter* | 0.0003 | 0.0023 | 0.0000 | 0.0000 |
| *Rikenellaceae;Unknown* | 0.0000 | 0.0000 | 0.0003 | 0.0015 |
| *Pseudoflavonifractor* | 0.0000 | 0.0000 | 0.0003 | 0.0015 |
| *Mesorhizobium* | 0.0000 | 0.0000 | 0.0003 | 0.0015 |
| *Alloscardovia* | 0.0002 | 0.0011 | 0.0000 | 0.0000 |
| *Bacillus* | 0.0002 | 0.0011 | 0.0000 | 0.0000 |
| *Facklamia* | 0.0002 | 0.0011 | 0.0000 | 0.0000 |
| *Tetragenococcus* | 0.0002 | 0.0011 | 0.0000 | 0.0000 |
| *Lactobacillaceae;Unknown* | 0.0002 | 0.0011 | 0.0000 | 0.0000 |
| *Selenomonas* | 0.0002 | 0.0011 | 0.0000 | 0.0000 |
