# Supplementary material for: Altered gut microbiota in Rett syndrome
Source: Microbiome. 2016 Jul 30;4:41. doi: 10.1186/s40168-016-0185-y (PMC4967335; doi:10.1186/s40168-016-0185-y)
Supplement: Additional file 9: Table S4. — Wilcoxon rank-sum test comparison of bacterial relative abundances at phylum and genus levels. (DOCX 22 kb) [file 40168_2016_185_MOESM9_ESM.docx]

**Supplementary Table 4:** Wilcoxon rank-sum test comparison of bacterial relative abundances at phylum and genus levels.

| *Phylum* | *p-value* | FDR |
| --- | --- | --- |
| *Actinobacteria* | 0.00005 | 0.00170 |
| *Bacteroidetes* | 0.00008 | 0.00213 |
| *Fusobacteria* | 0.02407 | 0.08647 |
| *Firmicutes* | 0.07023 | 0.20600 |
| *TM7* | 0.07675 | 0.21441 |
| *Lentisphaerae* | 0.11732 | 0.27065 |
| *Elusimicrobia* | 0.19816 | 0.36712 |
| *Cyanobacteria/Chloroplast* | 0.61100 | 0.73654 |
| *Unknown* | 0.72561 | 0.78848 |
| *Verrucomicrobia* | 0.82300 | 0.87787 |
| *Proteobacteria* | 1.00000 | 1.00000 |
| *Synergistetes* | 1.00000 | 1.00000 |

| *Genus* | *p-value* | FDR |
| --- | --- | --- |
| *Clostridiales;Unknown* | 0.00003 | 0.00152 |
| *Firmicutes;Unknown* | 0.00002 | 0.00152 |
| *Gemmiger* | 0.00001 | 0.00152 |
| *Barnesiella* | 0.00004 | 0.00170 |
| *Coprococcus* | 0.00008 | 0.00213 |
| *Bifidobacterium* | 0.00011 | 0.00222 |
| *Roseburia* | 0.00011 | 0.00222 |
| *Eggerthella* | 0.00016 | 0.00286 |
| *Ruminococcaceae;Unknown* | 0.00027 | 0.00434 |
| *Escherichia/Shigella* | 0.00033 | 0.00446 |
| *Veillonellaceae;Unknown* | 0.00031 | 0.00446 |
| *Enterococcus* | 0.00044 | 0.00518 |
| *Porphyromonadaceae;Unknown* | 0.00042 | 0.00518 |
| *Lactobacillales;Unknown* | 0.00048 | 0.00533 |
| *Erysipelotrichaceae incertae sedis* | 0.00082 | 0.00847 |
| *Alistipes* | 0.00104 | 0.01014 |
| *Odoribacter* | 0.00116 | 0.01076 |
| *Lachnospiraceae;Unknown* | 0.00129 | 0.01131 |
| *Butyricimonas* | 0.00137 | 0.01151 |
| *Bilophila* | 0.00156 | 0.01251 |
| *Enterobacteriaceae;Unknown* | 0.00169 | 0.01280 |
| *Haemophilus* | 0.00175 | 0.01280 |
| *Megasphaera* | 0.00197 | 0.01387 |
| *Ruminococcus* | 0.00273 | 0.01846 |
| *Clostridium XlVa* | 0.00309 | 0.02012 |
| *Lactonifactor* | 0.00322 | 0.02025 |
| *Oscillibacter* | 0.00382 | 0.02317 |
| *Eubacteriaceae;Unknown* | 0.00429 | 0.02515 |
| *Dialister* | 0.00451 | 0.02562 |
| *Coriobacteriaceae;Unknown* | 0.00547 | 0.03006 |
| *Faecalibacterium* | 0.00592 | 0.03159 |
| *Actinomyces* | 0.00658 | 0.03311 |
| *Bifidobacteriaceae;Unknown* | 0.00659 | 0.03311 |
| *Butyricicoccus* | 0.00700 | 0.03423 |
| *Sporobacter* | 0.00894 | 0.04251 |
| *Desulfovibrionaceae;Unknown* | 0.01314 | 0.05928 |
| *Parabacteroides* | 0.01291 | 0.05928 |
| *Granulicatella* | 0.01377 | 0.06060 |
| *Alphaproteobacteria;Unknown* | 0.01456 | 0.06102 |
| *Burkholderiales;Unknown* | 0.01441 | 0.06102 |
| *Paraprevotella* | 0.01553 | 0.06358 |
| *Bacteroidales;Unknown* | 0.01689 | 0.06756 |
| *Bacteroides* | 0.01804 | 0.07056 |
| *Sarcina* | 0.02012 | 0.07696 |
| *Lactobacillus* | 0.02140 | 0.08013 |
| *Fusobacterium* | 0.02407 | 0.08647 |
| *Parvimonas* | 0.02457 | 0.08648 |
| *Prevotella* | 0.02635 | 0.09094 |
| *Corynebacterium* | 0.03468 | 0.11737 |
| *Deltaproteobacteria;Unknown* | 0.03929 | 0.13047 |
| *Phascolarctobacterium* | 0.04454 | 0.14517 |
| *Anaerotruncus* | 0.05115 | 0.16368 |
| *Kocuria* | 0.05544 | 0.16824 |
| *Proteus* | 0.05543 | 0.16824 |
| *Staphylococcus* | 0.05544 | 0.16824 |
| *Allisonella* | 0.06077 | 0.18127 |
| *TM7 genera incertae sedis* | 0.07675 | 0.21441 |
| *Veillonella* | 0.07562 | 0.21441 |
| *Solobacterium* | 0.08268 | 0.22737 |
| *Peptostreptococcus* | 0.09151 | 0.24777 |
| *Bacteroidetes;Unknown* | 0.09792 | 0.25344 |
| *Dorea* | 0.09768 | 0.25344 |
| *Morganella* | 0.09518 | 0.25344 |
| *Anaerococcus* | 0.09997 | 0.25499 |
| *Actinobacteria;Unknown* | 0.11330 | 0.27065 |
| *Clostridium IV* | 0.11594 | 0.27065 |
| *Eubacterium* | 0.11708 | 0.27065 |
| *Gemella* | 0.11841 | 0.27065 |
| *Oxalobacter* | 0.11592 | 0.27065 |
| *Victivallis* | 0.11732 | 0.27065 |
| *Xylanibacter* | 0.10821 | 0.27065 |
| *Lactococcus* | 0.12012 | 0.27103 |
| *Anaeroglobus* | 0.12372 | 0.27563 |
| *Streptococcus* | 0.14862 | 0.32697 |
| *Actinomycetales;Unknown* | 0.15086 | 0.32779 |
| *Clostridiaceae 1;Unknown* | 0.16208 | 0.34631 |
| *Sutterella* | 0.16331 | 0.34631 |
| *Atopobium* | 0.19007 | 0.36712 |
| *Cardiobacterium* | 0.19816 | 0.36712 |
| *Elusimicrobium* | 0.19816 | 0.36712 |
| *Gordonia* | 0.19816 | 0.36712 |
| *Hydrogenoanaerobacterium* | 0.19816 | 0.36712 |
| *Mesorhizobium* | 0.19816 | 0.36712 |
| *Paraeggerthella* | 0.19816 | 0.36712 |
| *Parasutterella* | 0.18578 | 0.36712 |
| *Pseudoflavonifractor* | 0.19816 | 0.36712 |
| *Rikenellaceae;Unknown* | 0.19816 | 0.36712 |
| *Scardovia* | 0.18658 | 0.36712 |
| *Clostridium XlVb* | 0.21116 | 0.38713 |
| *Gordonibacter* | 0.24322 | 0.44130 |
| *Actinobaculum* | 0.28677 | 0.48033 |
| *Anaerovorax* | 0.26886 | 0.48033 |
| *Mobiluncus* | 0.27554 | 0.48033 |
| *Mogibacterium* | 0.28929 | 0.48033 |
| *Murdochiella* | 0.28677 | 0.48033 |
| *Peptococcus* | 0.27305 | 0.48033 |
| *Peptostreptococcaceae;Unknown* | 0.27728 | 0.48033 |
| *Pseudomonas* | 0.28677 | 0.48033 |
| *Pseudoramibacter* | 0.28677 | 0.48033 |
| *Megamonas* | 0.29292 | 0.48181 |
| *Proteobacteria;Unknown* | 0.29637 | 0.48297 |
| *Acidaminococcus* | 0.30026 | 0.48482 |
| *Olsenella* | 0.30607 | 0.48971 |
| *Varibaculum* | 0.33763 | 0.53535 |
| *Anaerofustis* | 0.35423 | 0.55665 |
| *Clostridium sensu stricto* | 0.36173 | 0.56340 |
| *Finegoldia* | 0.37028 | 0.57166 |
| *Erysipelotrichaceae;Unknown* | 0.37391 | 0.57224 |
| *Holdemania* | 0.38664 | 0.58662 |
| *Abiotrophia* | 0.46215 | 0.59371 |
| *Alloscardovia* | 0.46215 | 0.59371 |
| *Anaerofilum* | 0.40288 | 0.59371 |
| *Anaerostipes* | 0.40712 | 0.59371 |
| *Arcanobacterium* | 0.46215 | 0.59371 |
| *Bacillus* | 0.46215 | 0.59371 |
| *Clostridiales Incertae Sedis XIII;Unknown* | 0.46215 | 0.59371 |
| *Devosia* | 0.46215 | 0.59371 |
| *Enhydrobacter* | 0.46215 | 0.59371 |
| *Facklamia* | 0.46215 | 0.59371 |
| *Howardella* | 0.46215 | 0.59371 |
| *Lactobacillaceae;Unknown* | 0.46215 | 0.59371 |
| *Propionibacterium* | 0.43349 | 0.59371 |
| *Puniceicoccaceae;Unknown* | 0.46215 | 0.59371 |
| *Rhizobacter* | 0.46215 | 0.59371 |
| *Schwartzia* | 0.46215 | 0.59371 |
| *Selenomonas* | 0.46215 | 0.59371 |
| *Succiniclasticum* | 0.46215 | 0.59371 |
| *Tetragenococcus* | 0.46215 | 0.59371 |
| *Trueperella* | 0.46215 | 0.59371 |
| *Unknown* | 0.41255 | 0.59371 |
| *Prevotellaceae;Unknown* | 0.48211 | 0.61486 |
| *Turicibacter* | 0.48796 | 0.61785 |
| *Collinsella* | 0.52711 | 0.66265 |
| *Coprobacillus* | 0.53092 | 0.66271 |
| *Unknown* | 0.57398 | 0.71142 |
| *Flavonifractor* | 0.58619 | 0.72147 |
| *Acinetobacter* | 0.60160 | 0.73529 |
| *Streptophyta* | 0.61100 | 0.73654 |
| *Catenibacterium* | 0.62043 | 0.74282 |
| *Blautia* | 0.64352 | 0.75506 |
| *Pediococcus* | 0.63506 | 0.75506 |
| *Pyramidobacter* | 0.64239 | 0.75506 |
| *Desulfovibrionales;Unknown* | 0.65869 | 0.76774 |
| *Slackia* | 0.67791 | 0.78495 |
| *Cloacibacillus* | 0.72253 | 0.78848 |
| *Clostridia;Unknown* | 0.69455 | 0.78848 |
| *Comamonas* | 0.72253 | 0.78848 |
| *Lachnospiracea incertae sedis* | 0.72185 | 0.78848 |
| *Leclercia* | 0.70856 | 0.78848 |
| *Porphyromonas* | 0.72576 | 0.78848 |
| *Rikenella* | 0.69472 | 0.78848 |
| *Rothia* | 0.72253 | 0.78848 |
| *Unknown* | 0.72561 | 0.78848 |
| *Desulfovibrio* | 0.74359 | 0.80289 |
| *Akkermansia* | 0.80929 | 0.86850 |
| *Enterorhabdus* | 0.88503 | 0.93246 |
| *Sutterellaceae;Unknown* | 0.88503 | 0.93246 |
| *Weissella* | 0.89007 | 0.93246 |
| *Clostridium XVIII* | 0.90285 | 0.93566 |
| *Dysgonomonas* | 0.91439 | 0.93566 |
| *Mitsuokella* | 0.91439 | 0.93566 |
| *Pasteurellaceae;Unknown* | 0.91364 | 0.93566 |
| *Peptoniphilus* | 0.92302 | 0.93902 |
| *Clostridium XI* | 0.96755 | 0.97867 |
