# Supplementary material for: Altered gut microbiota in Rett syndrome
Source: Microbiome. 2016 Jul 30;4:41. doi: 10.1186/s40168-016-0185-y (PMC4967335; doi:10.1186/s40168-016-0185-y)
Supplement: Additional file 10: Figure S6. — Genus level relative abundances of the bacterial gut microbiota of healthy controls (HC) and Rett syndrome (RTT) subjects. (PDF 5470 kb) [file 40168_2016_185_MOESM10_ESM.pdf]

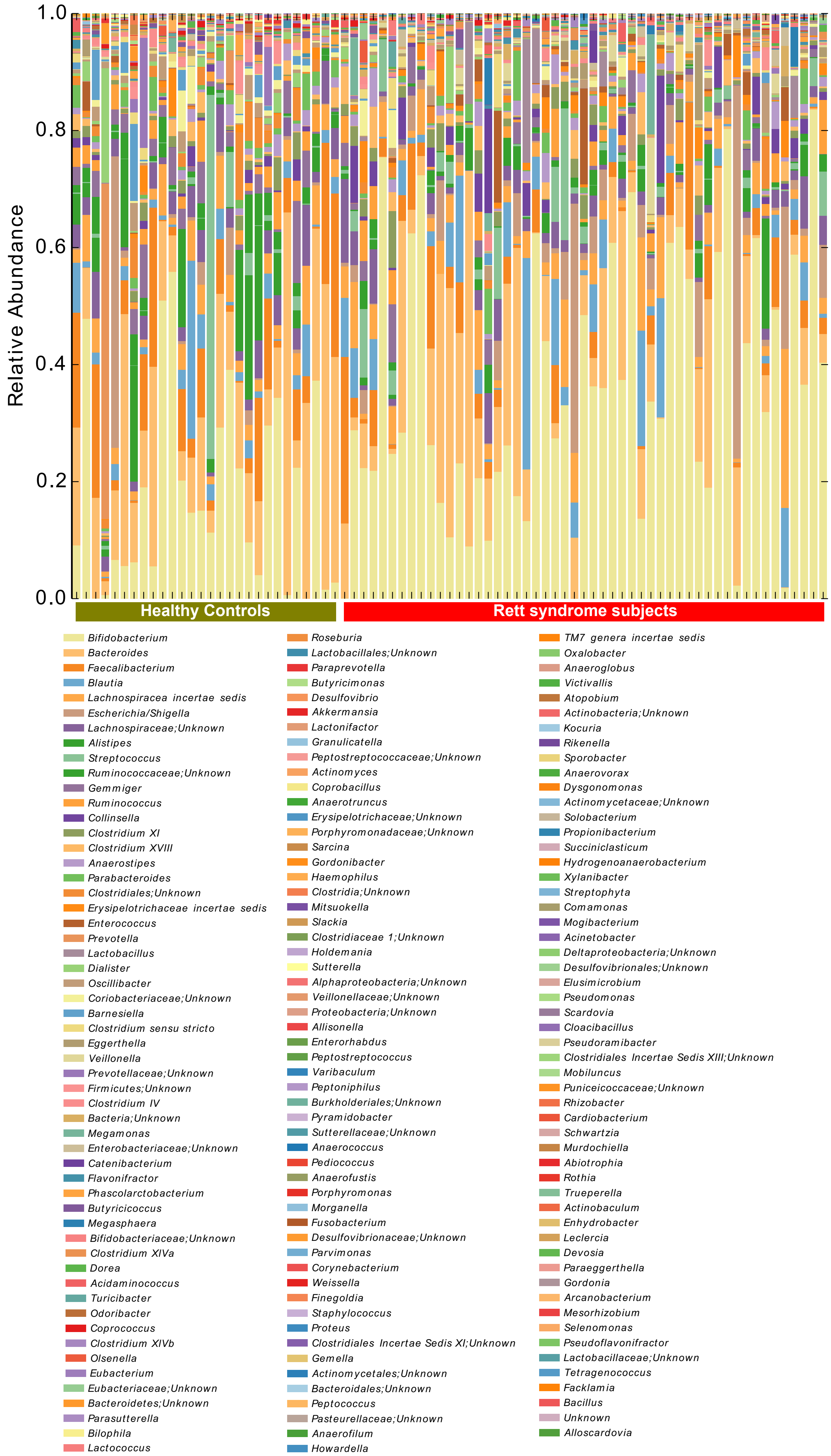

Relative Abundance
1.0
0.8
0.6
0.4
0.2
0.0
Healthy Controls
Rett syndrome subjects
Bifidobacterium
Bacteroides
Faecalibacterium
Blautia
Lachnospiracea incertae sedis
Escherichia/Shigella
Lachnospiraceae;Unknown
Alistipes
Streptococcus
Ruminococcaceae;Unknown
Gemmiger
Ruminococcus
Collinsella
Clostridium XI
Clostridium XVIII
Anaerostipes
Parabacteroides
Clostridiales;Unknown
Erysipelotrichaceae incertae sedis
Enterococcus
Prevotella
Lactobacillus
Dialister
Oscillibacter
Coriobacteriaceae;Unknown
Barnesiella
Clostridium sensu stricto
Eggerthella
Veillonella
Prevotellaceae;Unknown
Firmicutes;Unknown
Clostridium IV
Bacteria;Unknown
Megamonas
Enterobacteriaceae;Unknown
Catenibacterium
Flavonifractor
Phascolarctobacterium
Butyricicoccus
Megasphaera
Bifidobacteriaceae;Unknown
Clostridium XlVa
Dorea
Acidaminococcus
Turicibacter
Odoribacter
Coprococcus
Clostridium XlVb
Olsenella
Eubacterium
Eubacteriaceae;Unknown
Bacteroidetes;Unknown
Parasutterella
Bilophila
Lactococcus
Roseburia
Lactobacillales;Unknown
Paraprevotella
Butyricimonas
Desulfovibrio
Akkermansia
Lactonifactor
Granulicatella
Peptostreptococcaceae;Unknown
Actinomyces
Coprobacillus
Anaerotruncus
Erysipelotrichaceae;Unknown
Porphyromonadaceae;Unknown
Sarcina
Gordonibacter
Haemophilus
Clostridia;Unknown
Mitsuokella
Slackia
Clostridiaceae 1;Unknown
Holdemania
Sutterella
Alphaproteobacteria;Unknown
Veillonellaceae;Unknown
Proteobacteria;Unknown
Allisonella
Enterorhabdus
Peptostreptococcus
Varibaculum
Peptoniphilus
Burkholderiales;Unknown
Pyramidobacter
Sutterellaceae;Unknown
Anaerococcus
Pediococcus
Anaerofustis
Porphyromonas
Morganella
Fusobacterium
Desulfovibrionaceae;Unknown
Parvimonas
Corynebacterium
Weissella
Finegoldia
Staphylococcus
Proteus
Clostridiales Incertae Sedis XI;Unknown
Gemella
Actinomycetales;Unknown
Bacteroidales;Unknown
Peptococcus
Pasteurellaceae;Unknown
Anaerofilum
Howardella
TM7 genera incertae sedis
Oxalobacter
Anaeroglobus
Victivallis
Atopobium
Actinobacteria;Unknown
Kocuria
Rikenella
Sporobacter
Anaerovorax
Dysgonomonas
Actinomycetaceae;Unknown
Solobacterium
Propionibacterium
Succiniclasticum
Hydrogenoanaerobacterium
Xylanibacter
Streptophyta
Comamonas
Mogibacterium
Acinetobacter
Deltaproteobacteria;Unknown
Desulfovibrionales;Unknown
Elusimicrobium
Pseudomonas
Scardovia
Cloacibacillus
Pseudoramibacter
Clostridiales Incertae Sedis XIII;Unknown
Mobiluncus
Puniceicoccaceae;Unknown
Rhizobacter
Cardiobacterium
Schwartzia
Murdochiella
Abiotrophia
Rothia
Trueperella
Actinobaculum
Enhydrobacter
Leclercia
Devosia
Paraeggerthella
Gordonia
Arcanobacterium
Mesorhizobium
Selenomonas
Pseudoflavonifractor
Lactobacillaceae;Unknown
Tetragenococcus
Facklamia
Bacillus
Unknown
Alloscardovia
