# Supplementary material for: Altered gut microbiota in Rett syndrome
Source: Microbiome. 2016 Jul 30;4:41. doi: 10.1186/s40168-016-0185-y (PMC4967335; doi:10.1186/s40168-016-0185-y)
Supplement: Additional file 13: Table S5. — Statistics of the significantly different metabolic pathways (KEGG categories) inferred with PICRUSt in the gut microbiota of healthy controls (HC) and Rett syndrome (RTT) subjects (Welch’s t test, p < 0.05 FDR-corrected) from 16S rDNA data. (DOCX 26 kb) [file 40168_2016_185_MOESM13_ESM.docx]

**Supplementary Table 5:** Statistics of the significantly different metabolic pathways (KEGG categories) inferred with PICRUSt in the gut microbiota of healthy controls (HC) and Rett syndrome (RTT) subjects (Welch’s *t*-test, p<0.05 FDR-corrected) from 16S rDNA data.

| **KEGG categories** | **Healthy controls** | | **RTT patients** | | **p-values** | **p-adj** | **95.0% lower CI** | **95.0% upper CI** |
| --- | --- | --- | --- | --- | --- | --- | --- | --- |
|  | **mean rel. freq. (%)** | **std. dev. (%)** | **mean rel. freq. (%)** | **std. dev. (%)** |  |  |  |  |
| Phosphotransferase system (PTS) | 0.371433 | 0.123433 | 0.598653 | 0.258281 | 0.000002 | 0.000536 | -0.314180 | -0.140261 |
| Transporters | 6.808851 | 0.965836 | 7.889148 | 0.943105 | 0.000014 | 0.000887 | -1.534563 | -0.626031 |
| Chromosome | 1.575911 | 0.091076 | 1.479764 | 0.062469 | 0.000011 | 0.000936 | 0.057055 | 0.135238 |
| Restriction enzyme | 0.216111 | 0.043790 | 0.166141 | 0.042151 | 0.000009 | 0.000982 | 0.029472 | 0.070469 |
| Chaperones and folding catalysts | 1.008852 | 0.092890 | 0.911429 | 0.077359 | 0.000021 | 0.001145 | 0.055760 | 0.139086 |
| Transcription machinery | 0.976725 | 0.100412 | 0.867090 | 0.102835 | 0.000025 | 0.001186 | 0.061622 | 0.157648 |
| Lipid biosynthesis proteins | 0.579641 | 0.036582 | 0.534886 | 0.042453 | 0.000007 | 0.001227 | 0.026391 | 0.063119 |
| Ascorbate and aldarate metabolism | 0.098602 | 0.022726 | 0.128234 | 0.036042 | 0.000032 | 0.001324 | -0.042985 | -0.016279 |
| Plant-pathogen interaction | 0.140824 | 0.018886 | 0.120213 | 0.021061 | 0.000040 | 0.001456 | 0.011284 | 0.029937 |
| General function prediction only | 3.608275 | 0.137215 | 3.458786 | 0.155383 | 0.000045 | 0.001468 | 0.081304 | 0.217673 |
| Xylene degradation | 0.058345 | 0.019100 | 0.076697 | 0.017200 | 0.000101 | 0.001944 | -0.027109 | -0.009596 |
| ABC transporters | 3.178954 | 0.422589 | 3.605578 | 0.441775 | 0.000092 | 0.002010 | -0.630201 | -0.223046 |
| Protein processing in endoplasmic reticulum | 0.064067 | 0.015210 | 0.048903 | 0.015541 | 0.000099 | 0.002028 | 0.007897 | 0.022431 |
| Chagas disease (American trypanosomiasis) | 0.008754 | 0.008443 | 0.018124 | 0.011049 | 0.000084 | 0.002111 | -0.013845 | -0.004894 |
| Protein export | 0.603850 | 0.032683 | 0.571587 | 0.031658 | 0.000091 | 0.002125 | 0.016933 | 0.047595 |
| Transcription related proteins | 0.003437 | 0.002811 | 0.007184 | 0.005401 | 0.000145 | 0.002163 | -0.005612 | -0.001881 |
| Epithelial cell signaling in Helicobacter pylori infection | 0.094189 | 0.011868 | 0.082544 | 0.012551 | 0.000144 | 0.002253 | 0.005904 | 0.017387 |
| Chlorocyclohexane and chlorobenzene degradation | 0.011154 | 0.005722 | 0.017594 | 0.007702 | 0.000083 | 0.002257 | -0.009515 | -0.003365 |
| Energy metabolism | 0.869032 | 0.121532 | 0.750978 | 0.127371 | 0.000159 | 0.002265 | 0.059453 | 0.176656 |
| Drug metabolism - other enzymes | 0.335110 | 0.037774 | 0.302137 | 0.020652 | 0.000127 | 0.002322 | 0.017333 | 0.048613 |
| Prostate cancer | 0.040448 | 0.009196 | 0.031359 | 0.009916 | 0.000143 | 0.002348 | 0.004607 | 0.013571 |
| Dioxin degradation | 0.060495 | 0.020803 | 0.079556 | 0.016884 | 0.000139 | 0.002406 | -0.028330 | -0.009793 |
| African trypanosomiasis | 0.009944 | 0.008824 | 0.019585 | 0.011132 | 0.000083 | 0.002462 | -0.014238 | -0.005045 |
| Chloroalkane and chloroalkene degradation | 0.197584 | 0.035617 | 0.230084 | 0.031046 | 0.000182 | 0.002483 | -0.048675 | -0.016324 |
| Glycerophospholipid metabolism | 0.556524 | 0.045365 | 0.514331 | 0.043530 | 0.000197 | 0.002484 | 0.020979 | 0.063406 |
| Riboflavin metabolism | 0.211331 | 0.039616 | 0.176516 | 0.029317 | 0.000191 | 0.002507 | 0.017544 | 0.052085 |
| Ion channels | 0.017188 | 0.010138 | 0.026582 | 0.010465 | 0.000276 | 0.003348 | -0.014256 | -0.004533 |
| Drug metabolism - cytochrome P450 | 0.032544 | 0.015327 | 0.047231 | 0.017704 | 0.000300 | 0.003519 | -0.022366 | -0.007008 |
| Metabolism of xenobiotics by cytochrome P450 | 0.032463 | 0.015297 | 0.047071 | 0.017762 | 0.000322 | 0.003640 | -0.022289 | -0.006927 |
| Cytoskeleton proteins | 0.392817 | 0.047263 | 0.350158 | 0.048095 | 0.000361 | 0.003816 | 0.020111 | 0.065207 |
| Ubiquitin system | 0.013709 | 0.007738 | 0.021551 | 0.010444 | 0.000350 | 0.003828 | -0.012007 | -0.003677 |
| Progesterone-mediated oocyte maturation | 0.036074 | 0.007734 | 0.028931 | 0.008823 | 0.000446 | 0.004307 | 0.003287 | 0.010997 |
| Synthesis and degradation of ketone bodies | 0.021521 | 0.006037 | 0.016095 | 0.006313 | 0.000423 | 0.004333 | 0.002518 | 0.008335 |
| Prion diseases | 0.003041 | 0.001880 | 0.005617 | 0.004260 | 0.000479 | 0.004365 | -0.003981 | -0.001172 |
| Antigen processing and presentation | 0.036074 | 0.007734 | 0.028931 | 0.008823 | 0.000446 | 0.004437 | 0.003287 | 0.010997 |
| NOD-like receptor signaling pathway | 0.038380 | 0.009217 | 0.030182 | 0.009629 | 0.000478 | 0.004476 | 0.003759 | 0.012637 |
| Butirosin and neomycin biosynthesis | 0.076643 | 0.012314 | 0.065715 | 0.013611 | 0.000619 | 0.005491 | 0.004868 | 0.016987 |
| One carbon pool by folate | 0.639248 | 0.058686 | 0.593137 | 0.042400 | 0.000683 | 0.005897 | 0.020657 | 0.071563 |
| Flagellar assembly | 0.207395 | 0.095427 | 0.126996 | 0.094264 | 0.000723 | 0.006078 | 0.035341 | 0.125457 |
| Carbon fixation in photosynthetic organisms | 0.673432 | 0.036854 | 0.643658 | 0.032598 | 0.000811 | 0.006654 | 0.012967 | 0.046582 |
| Function unknown | 1.221045 | 0.106717 | 1.314148 | 0.124039 | 0.000931 | 0.007448 | -0.146709 | -0.039496 |
| Proximal tubule bicarbonate reclamation | 0.020030 | 0.008701 | 0.012757 | 0.009208 | 0.001010 | 0.007886 | 0.003063 | 0.011484 |
| Others | 0.927654 | 0.072299 | 0.983807 | 0.060567 | 0.001070 | 0.008159 | -0.088631 | -0.023676 |
| Polyketide sugar unit biosynthesis | 0.237731 | 0.030644 | 0.213450 | 0.030187 | 0.001377 | 0.010264 | 0.009825 | 0.038737 |
| Carbohydrate metabolism | 0.195214 | 0.030237 | 0.220358 | 0.034839 | 0.001486 | 0.010832 | -0.040277 | -0.010010 |
| Histidine metabolism | 0.690466 | 0.074480 | 0.637199 | 0.052650 | 0.001721 | 0.012269 | 0.021108 | 0.085426 |
| Inositol phosphate metabolism | 0.101559 | 0.012481 | 0.112350 | 0.016401 | 0.001789 | 0.012485 | -0.017420 | -0.004162 |
| Fatty acid metabolism | 0.258928 | 0.041966 | 0.294572 | 0.053866 | 0.001912 | 0.012801 | -0.057683 | -0.013604 |
| Proteasome | 0.052386 | 0.012189 | 0.062559 | 0.014996 | 0.001883 | 0.012868 | -0.016450 | -0.003896 |
| Ribosome biogenesis in eukaryotes | 0.055503 | 0.013070 | 0.066227 | 0.015763 | 0.002045 | 0.013417 | -0.017394 | -0.004053 |
| Translation factors | 0.552452 | 0.040026 | 0.524852 | 0.028837 | 0.002494 | 0.016043 | 0.010250 | 0.044950 |
| Base excision repair | 0.440707 | 0.021299 | 0.424864 | 0.021213 | 0.002622 | 0.016537 | 0.005758 | 0.025928 |
| Tyrosine metabolism | 0.360993 | 0.032997 | 0.388238 | 0.043420 | 0.002793 | 0.016967 | -0.044782 | -0.009707 |
| Polycyclic aromatic hydrocarbon degradation | 0.117622 | 0.012024 | 0.108404 | 0.013412 | 0.002870 | 0.017114 | 0.003280 | 0.015157 |
| Bacterial chemotaxis | 0.288587 | 0.078897 | 0.232275 | 0.071952 | 0.002990 | 0.017207 | 0.020004 | 0.092621 |
| Primary immunodeficiency | 0.048920 | 0.007695 | 0.055219 | 0.010062 | 0.002940 | 0.017222 | -0.010377 | -0.002222 |
| Transcription factors | 1.690215 | 0.177764 | 1.823132 | 0.183453 | 0.002786 | 0.017244 | -0.218151 | -0.047684 |
| Shigellosis | 0.000000 | 0.000000 | 0.000004 | 0.000010 | 0.003219 | 0.018207 | -0.000007 | -0.000002 |
| Pathways in cancer | 0.039025 | 0.008329 | 0.032571 | 0.010061 | 0.003486 | 0.019379 | 0.002200 | 0.010708 |
| Carbon fixation pathways in prokaryotes | 0.949869 | 0.081717 | 0.892225 | 0.077806 | 0.003703 | 0.020242 | 0.019527 | 0.095761 |
| Lipid metabolism | 0.123550 | 0.023902 | 0.104193 | 0.033699 | 0.004484 | 0.024113 | 0.006202 | 0.032512 |
| Bacterial motility proteins | 0.556595 | 0.189692 | 0.425357 | 0.184722 | 0.004626 | 0.024474 | 0.042100 | 0.220374 |
| Ribosome Biogenesis | 1.429153 | 0.078921 | 1.378516 | 0.061728 | 0.005296 | 0.027571 | 0.015793 | 0.085481 |
| Selenocompound metabolism | 0.387517 | 0.028658 | 0.409540 | 0.038965 | 0.005928 | 0.030379 | -0.037503 | -0.006542 |
| Glutathione metabolism | 0.194576 | 0.037021 | 0.220928 | 0.042683 | 0.006022 | 0.030386 | -0.044885 | -0.007818 |
| Membrane and intracellular structural molecules | 0.489043 | 0.137976 | 0.392971 | 0.152215 | 0.006243 | 0.031026 | 0.028225 | 0.163918 |
| Other transporters | 0.256346 | 0.029236 | 0.232426 | 0.045811 | 0.006605 | 0.031862 | 0.006862 | 0.040978 |
| Zeatin biosynthesis | 0.056130 | 0.008212 | 0.051131 | 0.005685 | 0.006582 | 0.032220 | 0.001468 | 0.008530 |
| Ethylbenzene degradation | 0.040695 | 0.014248 | 0.031044 | 0.015234 | 0.007055 | 0.033537 | 0.002729 | 0.016573 |
| Propanoate metabolism | 0.461379 | 0.036262 | 0.485831 | 0.040058 | 0.008005 | 0.035966 | -0.042292 | -0.006612 |
| Styrene degradation | 0.011358 | 0.005122 | 0.015654 | 0.008668 | 0.007786 | 0.035968 | -0.007425 | -0.001166 |
| Arginine and proline metabolism | 1.282244 | 0.086147 | 1.228878 | 0.071400 | 0.007691 | 0.036038 | 0.014776 | 0.091956 |
| Retinol metabolism | 0.035106 | 0.012620 | 0.044640 | 0.017861 | 0.007927 | 0.036114 | -0.016493 | -0.002574 |
| Glycosphingolipid biosynthesis - ganglio series | 0.069709 | 0.042315 | 0.042320 | 0.042644 | 0.008474 | 0.037561 | 0.007270 | 0.047508 |
| Insulin signaling pathway | 0.089897 | 0.011488 | 0.082731 | 0.011126 | 0.010051 | 0.042816 | 0.001778 | 0.012555 |
| Tuberculosis | 0.155036 | 0.012125 | 0.147335 | 0.012348 | 0.009963 | 0.043000 | 0.001914 | 0.013487 |
| Basal transcription factors | 0.002927 | 0.003096 | 0.001199 | 0.001756 | 0.009916 | 0.043365 | 0.000439 | 0.003016 |
| Glutamatergic synapse | 0.116836 | 0.011225 | 0.109912 | 0.010650 | 0.010393 | 0.043703 | 0.001694 | 0.012154 |
| Biosynthesis of ansamycins | 0.112961 | 0.011872 | 0.104325 | 0.017073 | 0.011067 | 0.045948 | 0.002033 | 0.015239 |
