# Supplementary material for: Altered gut microbiota in Rett syndrome
Source: Microbiome. 2016 Jul 30;4:41. doi: 10.1186/s40168-016-0185-y (PMC4967335; doi:10.1186/s40168-016-0185-y)
Supplement: Additional file 15: Figure S10. — Genus level relative abundances of the fungal gut microbiota of healthy controls (HC) and Rett syndrome (RTT) subjects. (PDF 2981 kb) [file 40168_2016_185_MOESM15_ESM.pdf]

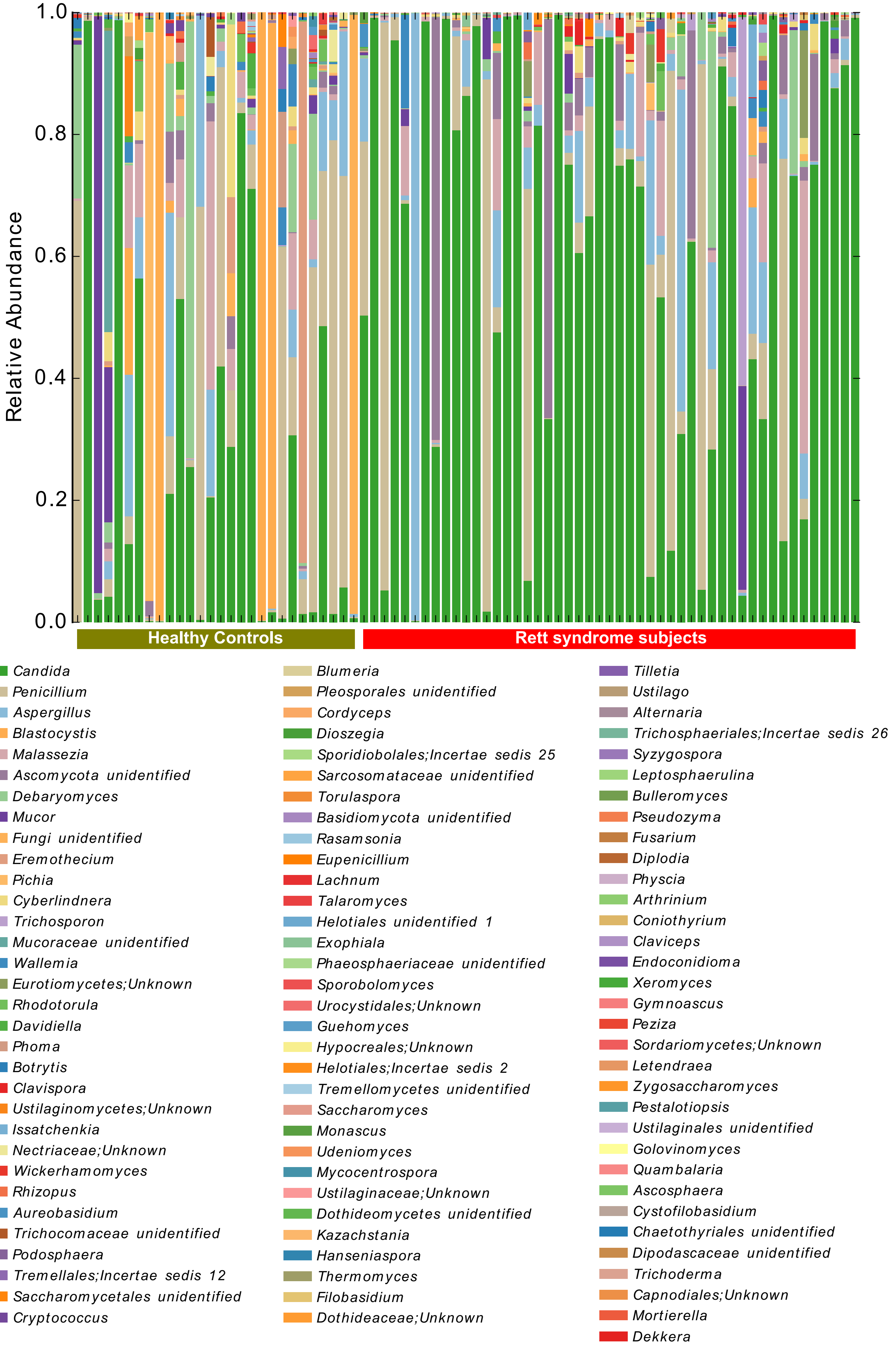

Relative Abundance
1.0
0.8
0.6
0.4
0.2
0.0
Healthy Controls
Rett syndrome subjects
Candida
Penicillium
Aspergillus
Blastocystis
Malassezia
Ascomycota unidentified
Debaryomyces
Mucor
Fungi unidentified
Eremothecium
Pichia
Cyberlindnera
Trichosporon
Mucoraceae unidentified
Wallemia
Eurotiomycetes;Unknown
Rhodotorula
Davidiella
Phoma
Botrytis
Clavispora
Ustilaginomycetes;Unknown
Issatchenkia
Nectriaceae;Unknown
Wickerhamomyces
Rhizopus
Aureobasidium
Trichocomaceae unidentified
Podosphaera
Tremellales;Incertae sedis 12
Saccharomycetales unidentified
Cryptococcus
Blumeria
Pleosporales unidentified
Cordyceps
Dioszegia
Sporidiobolales;Incertae sedis 25
Sarcosomataceae unidentified
Torulaspora
Basidiomycota unidentified
Rasamsonia
Eupenicillium
Lachnum
Talaromyces
Helotiales unidentified 1
Exophiala
Phaeosphaeriaceae unidentified
Sporobolomyces
Urocystidales;Unknown
Guehomyces
Hypocreales;Unknown
Helotiales;Incertae sedis 2
Tremellomycetes unidentified
Saccharomyces
Monascus
Udeniomyces
Mycocentrospora
Ustilaginaceae;Unknown
Dothideomycetes unidentified
Kazachstania
Hanseniaspora
Thermomyces
Filobasidium
Dothideaceae;Unknown
Tilletia
Ustilago
Alternaria
Trichosphaeriales;Incertae sedis 26
Syzygospora
Leptosphaerulina
Bulleromyces
Pseudozyma
Fusarium
Diplodia
Physcia
Arthrinium
Coniothyrium
Claviceps
Endoconidioma
Xeromyces
Gymnoascus
Peziza
Sordariomycetes;Unknown
Letendraea
Zygosaccharomyces
Pestalotiopsis
Ustilaginales unidentified
Golovinomyces
Quambalaria
Ascosphaera
Cystofilobasidium
Chaetothyriales unidentified
Dipodascaceae unidentified
Trichoderma
Capnodiales;Unknown
Mortierella
Dekkera
