# Supplementary material for: Altered gut microbiota in Rett syndrome
Source: Microbiome. 2016 Jul 30;4:41. doi: 10.1186/s40168-016-0185-y (PMC4967335; doi:10.1186/s40168-016-0185-y)
Supplement: Additional file 19. — MICCA pipelines used for the analysis of the 16S and ITS1 datasets. (PDF 7 kb) [file 40168_2016_185_MOESM19_ESM.pdf]

Supplementary Material: MICCA pipelines used for the analysis of the 16S and ITS1 datasets

MICCA (v 0.1) (http://www.micca.org)

16S dataset pipeline

micca-preproc -f TCCTACGGGAGGCAGCAG -r TGTGCGGGCCCCCGTCAATT -O 16 -q 20 -l 400 fastq/*.fastq -o preprocessed

micca-otu-denovo preprocessed/*.fastq -s 0.97 -d -l 200 -c -o otus

micca-phylogeny otus/representatives.fasta -a template -o otus/phylo --template-min-perc 75 --template-file ~/greengenes_2013_05/rep_set_aligned/97_otus.fasta

micca-midpoint-root otus/phylo/tree.tre otus/phylo/tree_rooted.tre

ITS1 dataset pipeline

micca-preproc -f GTAAAAGTCGTAACAAGGTTTC -r GTTCAAAGAYTCGATGATTCAC -O 18 -q 15 -l 150 fastq/*.fastq -o preprocessed

micca-otu-denovo preprocessed/*.fastq -s 0.97 -c -d -l 140 -o otus -t rdp --rdp-gene fungalits_unite --rdp max-memory 2000

micca-phylogeny otus/representatives.fasta -a denovo_tcoffe --tcoffe-num-threads 1 -o otus/phylo

micca-midpoint-root otus/phylo/tree.tre otus/phylo/tree_rooted.tre
